# Understanding Merkel Cell Carcinoma: Pathogenic Signaling, Extracellular Matrix Dynamics, and Novel Treatment Approaches

**DOI:** 10.3390/cancers17071212

**Published:** 2025-04-02

**Authors:** Maria Konstantaraki, Aikaterini Berdiaki, Monica Neagu, Sabina Zurac, Konstantinos Krasagakis, Dragana Nikitovic

**Affiliations:** 1 Department of Histology-Embryology, Medical School, University of Crete, 71003 Heraklion, Greece; mariakonstantar@gmail.com (M.K.); berdiaki@uoc.gr (A.B.); 2 Dermatology Department, University Hospital of Heraklion, 71110 Heraklion, Greece; krasagak@uoc.gr; 3 Immunology Laboratory, “Victor Babes” National Institute of Pathology, 99-101 Splaiul Independenței, 050096 Bucharest, Romania; neagu.monica@gmail.com; 4 Pathology Department, Colentina Clinical Hospital, 19-21 Sos Stefan Cel Mare, 020125 Bucharest, Romania; sabina_zurac@yahoo.com; 5 Faculty of Dentistry, University of Medicine and Pharmacy, 8 Eroilor Sanitari Boulevard, 050474 Bucharest, Romania

**Keywords:** Merkel cell carcinoma, extracellular matrix, signaling pathways, immunology, targeted molecular treatment

## Abstract

Merkel cell carcinoma (MCC) is a rare but aggressive neuroendocrine skin cancer, driven by either Merkel cell polyomavirus (MCPyV) integration or ultraviolet (UV)-induced mutations. In MCPyV-positive tumors, viral T antigens inactivate tumor suppressors pRb and p53, while virus-negative MCCs harbor UV-induced mutations that activate similar oncogenic pathways. Key signaling cascades, including PI3K/AKT/mTOR and MAPK, support tumor proliferation, survival, and resistance to apoptosis. Histologically, MCC consists of small round blue cells with neuroendocrine features, high mitotic rate, and necrosis. The tumor microenvironment (TME) plays a central role in disease progression and immune escape. It comprises a mix of tumor-associated macrophages, regulatory and cytotoxic T cells, and elevated expression of immune checkpoint molecules such as PD-L1, contributing to an immunosuppressive niche. The extracellular matrix (ECM) within the TME is rich in proteoglycans, collagens, and matrix metalloproteinases (MMPs), facilitating tumor cell adhesion, invasion, and interaction with stromal and immune cells. ECM remodeling and integrin-mediated signaling further promote immune evasion and therapy resistance. Although immune checkpoint inhibitors targeting PD-1/PD-L1 have shown promise in treating MCC, resistance remains a major hurdle. Therapeutic strategies that concurrently target the TME—through inhibition of ECM components, MMPs, or integrin signaling—may enhance immune responses and improve clinical outcomes.

## Simple Summary

Merkel cell carcinoma is an aggressive neuroendocrine malignancy with a globally rising incidence. Progress in exploring its pathogenetic mechanisms has revealed various crucial pathways leading to the development of this cancer, although its exact mechanism has not yet been scrutinized. Treatment modalities have been enriched over the last few decades but challenges due to resistance and poor response remain. This study aims to outline the current data on the pathogenesis of this disease, including the role of signaling mechanisms, the extracellular matrix, and the immunology of this cancer. Treatment options, including standard therapeutical approaches and immunotherapies, will be delineated, along with possible future targeted molecular treatments, arising from the mechanisms involved in the pathogenesis of Merkel cell carcinoma.

## 1. Introduction

Merkel cell carcinoma (MCC) is a rare but lethal primary neuroendocrine cancer mainly occurring in elderly Caucasian individuals, with a constantly rising incidence globally. As recent epidemiological works reflect, although the incidence rates of MCC seem to vary among different regions of the world, there is an overall exponential increase [1,2,3]. This increase might be associated with a genuine rise in the number of MCC cases diagnosed, as well as improved access to healthcare, enhanced diagnostic approaches, and increased awareness among healthcare professionals. The overall increased global life expectancy, along with the enhanced prognosis of patients who suffer from multiple comorbidities and immunosuppression, also affects the total number of MCCs diagnosed. The role of the immune system seems to have a growing effect on the growth of several malignancies, including MCC, since research reveals that a favorable prognosis is associated with immune response genes [4]. Although MCC occurs at a significantly lower incidence than melanoma [1], it is twice as lethal due to its high migratory and metastatic potential [5]. With an estimated mortality rate of 33–46%, MCC has become the second leading cause of skin cancer-related deaths [6].

Merkel cell polyomavirus (MCPyV) and exposure to ultraviolet (UV) radiation are significant risk factors for developing MCC. Based on the presence of MCPyV, two subtypes of MCC are recognized: MCPyV-positive MCC and MCPyV-negative MCC. MCPyV is a common virus discovered in 2008. It infects most people during childhood and is present in 8 out of 10 cases of MCC, thus reinforcing its correlation with the carcinogenesis of this tumor. Although human infection with MCPyV is ubiquitous, it remains asymptomatic in most cases.

Chronic unprotected exposure to UV radiation also clearly correlates with MCC, and it remains a further key risk factor, as MCPyV-negative MCCs exhibit a high tumor mutational burden [7] and UV-induced DNA damage [8]. Therefore, it is indicated that MCC pathogenesis can be classified into two molecularly distinct etiologies: virus-induced and UV-induced. Although the prognostic significance of the viral status of MCC has not yet been clarified [9], there seems to be a favorable overall prognosis in MCPyV-positive MCCs [10]. A study that included 114 MCC tissue samples showed a threefold lower 5-year survival in the MCPyV DNA–negative tumors [11]. This could correlate to the findings that virus-negative MCC exhibits a significantly higher overall mutational rate than virus-positive MCC [8,12,13].

MCCs appear as painless red-violet nodules of the skin with a smooth surface. They are predominantly found in sun-exposed regions of the body, i.e., mainly the head and neck but also the trunk and limbs [14,15,16]. MCC, via lymphatic and hematogenous pathways, spreads to regional lymph nodes, the liver, lungs, bone, and brain; metastasis to lymph nodes is associated with a poor prognosis [16,17]. Further risk factors for MCC include immunosuppression due to a medical condition or specific immunosuppressant treatments, as well as advanced age, with the incidence of MCC increasing among individuals over 50–65 years old. Prognostication of MCC is performed using the consensus 8th edition American Joint Cancer Committee (AJCC) staging system, which uses the classical Tumor (T), regional lymph node (N), and distant metastasis (M) staging approach. However, the need for optimized staging tools is emerging [18,19]. Treatment of MCC remains challenging although efforts are made to ensure a structured management approach, including the latest agents available. The majority of patients affected by MCC are typically elderly, who usually suffer from several comorbidities and/or are immunosuppressed, characteristics that by definition limit the tolerance for standard available treatments, such as surgery or chemotherapy.

Although it likely plays a significant role in early diagnosis and targeted treatment of MCC, the cell of origin of this tumor remains unknown. As there are two subtypes of MCCs with vast genetic differences detected between them, recent studies suggest that MCPyV-positive and MCPyV-negative MCCs may evolve from two distinct cells of origin Specifically, the MCPyV-negative MCC is indicated to derive from epidermal keratinocytes whereas the MCPyV-positive MCC is suggested to originate from dermal fibroblasts, hence making MCC the first known cancer developing from separate germ layers [20].

## 2. Tumor Microenvironment/Tumor Niche

The unique and dynamic interactions between Merkel cell carcinoma and its tumor microenvironment (TME) significantly contribute to the cancer’s progression and therapeutic resistance. A deeper understanding of the extracellular matrix (ECM) cues within the MCC TME is critical for elucidating the mechanisms underlying tumor growth and identifying novel therapeutic targets.

Merkel cell carcinoma (MCC) is a rare but aggressive neuroendocrine skin cancer characterized by rapid growth, high propensity for metastasis, and poor prognosis in advanced stages. MCC cells engage in unique and dynamic interactions with their tumor microenvironment (TME), significantly contributing to this cancer’s progression and therapeutic resistance. A deeper understanding of the extracellular matrix (ECM) cues within the MCC TME is critical for elucidating the mechanisms underlying tumor growth and identifying novel therapeutic targets. The TME of MCC comprises a complex milieu composed of various cellular and non-cellular components, including immune cells, fibroblasts, endothelial cells, and ECM molecules. Indeed, the interaction between tumor cells and these components creates a supportive niche that facilitates MCC survival, invasion, and metastasis. Indeed, the leading players determining the TME of MCC are the immune cell landscape, cancer-associated fibroblasts (CAFs), endothelial cells and angiogenesis, and the components of the ECM [21,22,23,24].

### 2.1. Role of the Extracellular Matrix in Merkel Cell Carcinoma

The extracellular matrix (ECM) is a complex network of proteins and carbohydrates that provides structural support to cells and affects various cellular processes such as adhesion, migration, and signaling [25]. Additionally, it holds a well-known role in all stages of tumor progression, offering a constantly adapting and supportive environment for cancer cells to grow, invade, and metastasize [26,27]. Furthermore, research has lately focused on the ECM’s structural and functional effects on tumor invasion [28]. Although this remains an understudied field in MCC, evidence suggests that this tumor’s aggressiveness could be related to alterations in the ECM and the signaling pathways that mediate interactions between MCC and ECM components. Recent findings showing that cancer-associated fibroblasts exert proangiogenic activity in MCC corroborate this [21,29]. 

Indeed, the ECM is a critical regulator of the cancer cell TME, influencing cellular behavior through biochemical and mechanical cues. The composition, organization, and stiffness of the ECM are dynamically regulated during cancer progression [30]. Thus, in the case of keratinocyte-derived carcinomas, the ECM is rich in glycosaminoglycans (GAGs), proteoglycans (PGs), and fibrous proteins such as collagen and fibronectin [27]. Specific ECM molecules, including hyaluronan, promote keratinocyte-derived carcinoma cell proliferation, migration, and immune evasion [27]. Matrix remodeling enzymes, such as MMPs and lysyl oxidase (LOX), alter ECM integrity and stiffness, enhancing tumor cell motility and invasiveness [31]. Collagen crosslinking mediated by LOX increases ECM stiffness, activating integrin signaling pathways. Data suggest that fibronectin overexpression in MCC is associated with increased tumor aggressiveness and resistance to therapy [32].

Laurito et al. suggested that regardless of the MCC subtype, size, or anatomical location, the ECM in primary MCCs exhibits extensive alterations in collagen fibers. Specifically, in a study that included 11 immunocompetent adults with MCC, the peritumoral stroma was compared with normal dermis, and this evaluation exhibited minor normal collagen density, minor collagen fibers’ diameter, lower values of entropy, and contrast in the peritumoral space. These dissimilarities were not evident among MCCs with favorable and unfavorable prognostic factors [23].

Koljonen et al. described enhanced Tenascin-C (Tn-C) expression, which correlated with greater tumor size and the invasive region of MCC [33]. Tn-C is a glycoprotein encoded by the TNC gene, expressed in the ECM of traumatized or developing tissues. It generally regulates cell behavior via interactions with cell surface receptors such as integrins and TLR4 and determines integrin signaling. Increased expression of Tn-C has already been correlated with various malignancies and tumor growth [34]. Moreover, interactions between Merkel cell carcinoma cells and extracellular matrix components, mediated by cell surface receptors such as integrins, can activate signaling pathways that promote cell survival, proliferation, and invasion [35,36].

Integrins are significant ECM components and act as cellular receptors. They contribute to various aspects of cellular functions and promote cell growth, survival, and proliferation. Studying the microenvironment of a cancer cell has shown that the dysregulation of these signaling pathways has been determined in cancer [37]. Moreover, it may contribute to the aggressive behavior of MCC tumors, although this remains quite an understudied field. In MCPyV-positive MCCs, minor tumor antigens (STs) seem to contribute to tumorigenesis significantly. Interactions between the MCPyV ST and PP4C induce integrin β1 dephosphorylation and thus promote cell motility. Additionally, integrins are crucial in filopodia formation by the MCPyV ST, further promoting MCC cell motility and metastasis [35]. In addition to those ECM components, studies have shown an increased expression of matrix metalloproteinases (MMPs), which induce matrix remodeling and may support the MCPyV infection of human dermal fibroblasts [38]. Increased expression of MMP-1, MMP-3, MMP-7, MMP-10, MMP-26, and MMP-10/2 has been associated with MCC’s aggressiveness and correlated with a worse prognosis [39,40,41]. Accordingly, expression of TIMP-3 (which not only has inhibitory functions against MMPs but also seems to affect tumor progression) was found in over 90% of MCC tumoral cells [41].

Changes in ECM organization can be extrapolated as mechanotransduction—the process by which cells sense and respond to the mechanical properties of the ECM. Indeed, this seems to be a key feature of MCC progression [42]. Increased ECM stiffness, mediated by crosslinking enzymes and fibroblast contraction, activates integrin signaling and downstream pathways such as focal adhesion kinase (FAK) and Yes-associated protein (YAP) [30]. These signals drive MCC cell proliferation, survival, and migration. Mechanotransduction also influences nuclear dynamics, altering gene expression profiles that support tumor progression.

The ECM can modulate immune responses within the tumor microenvironment. Altered ECM components may create a physical barrier to immune cell infiltration or present signals that suppress immune activity, contributing to immune evasion in MCC [43]. The ECM also plays a pivotal role in modulating immune cell recruitment and function within the MCC TME. Altered ECM components may create a physical barrier to immune cell infiltration or present signals that suppress immune activity, contributing to immune evasion in MCC [43]. The ECM also plays a pivotal role in modulating immune cell recruitment and function within the MCC TME.

Understanding these signaling pathways and ECM-derived cues in MCC provides insights into the molecular mechanisms driving tumor progression and offers potential avenues for targeted therapeutic interventions.

### 2.2. Cancer-Associated Fibroblasts (CAFs)

CAFs are activated fibroblasts with a mesenchymal lineage associated with cancer. These cells also secrete matrix metalloproteinases (MMPs), proteoglycans, and glycoproteins that degrade and restructure the ECM, facilitating tumor cell invasion and metastasis [44]. They are generally a key component of the TME, exerting multiple protumorigenic functions, and exhibit notable heterogeneity, which limits their molecular targeting. Fibroblasts, epithelial cells, endothelial cells, cancer stem cells, adipocytes, pericytes, or stellate cells are some of the assumed precursors of CAFs, suggesting a possible correlation between their origin and functional heterogeneity [45]. A significant number of various tumor-promoting secretory factors, including TGFβ, PDGF, FGF, hepatocyte growth factor (HGF), VEGF, tumor necrosis factor α (TNFα), interferon-γ (IFNγ), CXCL12, IL-6, connective tissue growth factor (CTGFβ), EGF, growth arrest-specific protein 6 (GAS6), galectin-1, secreted frizzled-related protein 1 (SFRP1), sonic hedgehog protein (SHH), and bone morphogenetic protein (BMP) are secreted by CAFs [45]. Furthermore, CAFs promote tumor growth and metastasis via secretion of IL-6, causing the endothelial–mesenchymal transition, a key feature of metastatic cancer cells [24].

CAFs are key mediators of ECM remodeling in the MCC TME. Until recently, the function of CAFs in MCC had not been deeply investigated. Fan et al. demonstrated that CAFs in the stroma of MCC lesions exhibit a heterogeneity associated with a spectrum of polarization toward a CAF phenotype, as revealed by immunohistochemistry-based staining and scRNAseq. The functional effect of MCC-derived factors on fibroblasts was investigated in a series of experiments, where MCC cells were co-cultured with fibroblasts under different conditions, showing that fibroblasts were polarized toward a CAF phenotype by MCC-derived factors. The same study demonstrated that miR-375, which is horizontally transferred from MCC cells to fibroblasts and promotes intracellular communication, sufficiently causes fibroblast polarization by inhibiting RBPJ and p53 [24].

In a study with MCC specimens from 20 patients, 50% of the patients exhibited increased IL6+CAFs, while high IL6+CAFs were associated with poor prognosis. IL6 is a known cytokine in the TME, affecting various crucial cellular components of the tumor immune microenvironment. The fibrotic microenvironment rich in IL6+CAFs was associated with decreased high intratumoral CD8+ and CD4+TILs, which normally exert an anti-tumor effect, and was hence correlated with a poorer prognosis [22].

In MCC, CAFs have been shown to promote angiogenesis and tumor growth through ECM remodeling. Albertini et al. investigated the contribution of MCC-derived CAFs to angiogenesis. Subcutaneously co-injecting MCC-patient-derived CAFs along with MCC MKL-1 cells into severe combined immunodeficient mice led to significant tumor growth and metastasis, with characteristic human CAF-dense areas. Furthermore, they demonstrated that the angiogenesis induced by CAFs in MCC is mediated by the aminopeptidase A/angiotensin II and III/angiotensin II type 1 receptor axis, while the chemical inhibition of this pathway results in decreased CAF-induced angiogenesis [21].

### 2.3. Endothelial Cells and Angiogenesis

MCC tumors exhibit high vascular density, supported by pro-angiogenic factors such as vascular endothelial growth factor (VEGF) [21,32]. Hypoxia-inducible factors (HIFs) are also elevated in MCC, driving the expression of VEGF and other pro-angiogenic mediators [1], creating a pro-angiogenic niche that sustains tumor growth and provides a route for metastatic dissemination. Albertini et al. showed that the extent of angiogenesis varies among different MCC samples. At the same time, the volume of αSMA-1+ cells present in the tumor stroma is positively correlated with the vessel diameter [21].

The role of endothelial cells in MCC was investigated by Aung et al., showing that colocalized CD31 (endothelial marker) and B7-H3 (immune-regulatory protein) expression is correlated with increased tumor size, greater tumor depth, invasion, lymphovascular invasion, and invasion beyond the skin and an overall poorer prognosis in primary MCCs. In the same study, it was also highlighted that B7-H3 expression in the endothelial cells of primary MCCs possibly leads to vascular proliferation via activating proangiogenic procedures [46]. Moreover, a study that included 21 primary MCC tumor samples revealed positive staining for VEGFR-2 in the endothelial cells of the intratumoral vessels in 80% of them, with cytoplasmic patterns. VEGFR-2, a proangiogenic marker, was expressed in seven out of eight metastatic tumors. A positive correlation between tumor size and expression suggests a relationship between MCC neoangiogenesis and tumor development. In this context, it can be hypothesized that limiting angiogenesis may serve as an attenuating factor in MCCs [47].

**Figure 1:**
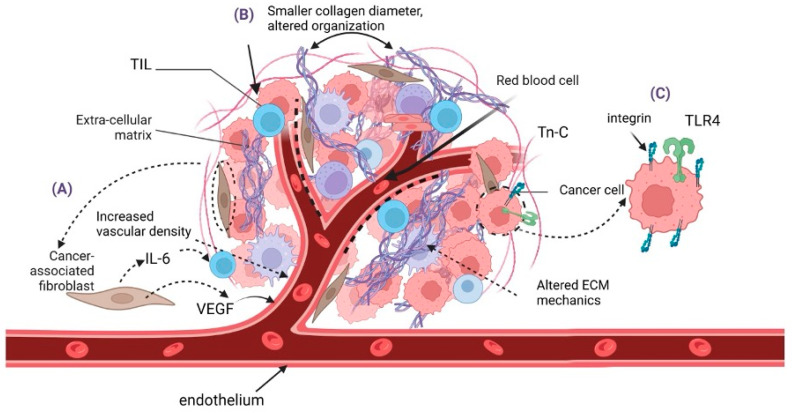
Tumor microenvironment (TME)-derived cues in MCC pathogenesis. (**A**) Cancer-associated fibroblasts (CAFs) in the MCC stroma display heterogeneity and promote ECM remodeling, angiogenesis, and tumor growth by secreting pro-angiogenic factors such as VEGF. (**B**) The MCC TME exhibits an altered collagen fiber density and diameter compared to normal dermis. Elevated tenascin-C (Tn-C) expression correlates with larger tumor size, interacting with integrins and TLR4 to influence signaling pathways. (**C**) Integrins, particularly β1 and β5, contribute to MCC progression. Created in BioRender. Nikitovic, D. (2025) https://BioRender.com/q23q581 (accessed on 24 March 2025).

Additionally, tumor endothelial cells can contribute to immune evasion by expressing PD-L1 and producing chemokines that recruit immunosuppressive cells. Angiogenesis inhibitors, such as bevacizumab, are being explored in combination with immune checkpoint inhibitors to target this aspect of the TME [48]. The key ECM-derived cues in MCC pathogenesis are depicted in Figure 1.

## 3. Signaling Mechanisms Involved in MCC Progression

The progression of this aggressive neuroendocrine skin cancer is characterized by complex signaling pathways that facilitate tumorigenesis and progression. Notably, tumor-intrinsic signaling and the tumor microenvironment are functionally interconnected and cannot be meaningfully separated, as highlighted below. However, understanding the specific involved signaling mechanisms is crucial for developing targeted therapies. However, the involved signaling pathways are understudied in MCC. Table 1 summarizes the below-discussed known signaling pathways involved in MCC.

### 3.1. PI3K/AKT/mTOR Pathway

The phosphoinositide 3-kinase (PI3K) signaling pathway is crucial in regulating cell growth, survival, metabolism, and proliferation. Activated by receptor tyrosine kinases (RTKs) and G-protein-coupled receptors (GPCRs), PI3K phosphorylates phosphatidylinositol-4,5-bisphosphate (PIP2) to generate phosphatidylinositol-3,4,5-trisphosphate (PIP3), which recruits downstream effectors such as AKT. This activation promotes cell survival and inhibits apoptosis through targets like mTOR and FOXO transcription factors. Dysregulation of PI3K signaling, often through mutations in PIK3CA, loss of PTEN, or hyperactivation of AKT, is implicated in various cancers, including breast, ovarian, and colorectal cancers, as well as cutaneous malignancies [75,76]. PI3K/Akt and mTORC1 affect tumor progression and contribute to the energy storage of cancer cells. Specifically, the upregulation of Akt results in the enhanced phosphorylation of target genes and proteins and hence blocks the process of apoptosis and autophagy, contributing to solid tumor growth [77]. Furthermore, studies have demonstrated that activation of mTOR dysregulates the normal cell cycle, by reducing autophagy and cell death [78]. Consequently, PI3K inhibitors are being explored as therapeutic strategies in oncology [79].

Regarding cutaneous malignancies, where UV exposure is a well-established risk factor in most of them, studies have shown that UVB and UVC exposure results in PI3K/AKT/mTOR pathway activation [80]. Specifically in melanoma, upregulation of PI3K correlated with a loss of PTEN, which is the negative regulator of the PR3K pathway, has been described in almost 50% of all cases [81,82]. Further immunochemistry studies showed overexpression of AKT in 60% of melanomas, which is an uncommon finding among dysplastic nevi [83], while elevated AKT has been associated with advanced-stage melanoma [84].

The PI3K/AKT/mTOR pathway is frequently activated in MCC cells, promoting their survival, growth, and proliferation. This activation occurs in both Merkel cell polyomavirus (MCPyV)-positive and MCPyV-negative MCCs, though the underlying mechanisms may differ. In MCPyV-negative MCCs, activating mutations in PIK3CA, the gene encoding the p110α catalytic subunit of PI3K [49], have been identified, leading to constitutive pathway activation. Indeed, Hafner et al., performing immunohistochemical analysis of 41 tumor tissues and 9 MCC cell lines, identified AKT phosphorylation at threonine 308 in most samples [50]. Likewise, Nardi et al. suggested that the activation of the PI3K pathway may contribute to tumorigenesis in a subset of MCC and that screening for PIK3CA mutations could help identify patients who might benefit from PI3K pathway inhibitors [51]. Iwasaki et al. indicated that the activation of most Akt/mTOR/4E-BP1 pathway signals does not significantly differ between MCPyV-positive and MCPyV-negative MCCs despite potential differences in their tumorigenesis. Notably, MCPyV-negative MCCs exhibit a higher p-Akt (T308) activation frequency. Consequently, targeting specific components of the Akt/mTOR/4E-BP1 pathway may offer novel therapeutic strategies for MCC, irrespective of MCPyV infection status [52]. On the other hand, it has been suggested that pathway activation in MCPyV-positive MCCs may be triggered via alternative mechanisms, such as viral oncoprotein interactions [51]. A recent study analyzed RNA sequencing data from formalin-fixed paraffin-embedded tissue samples of 102 MCC patients to identify differentially expressed genes between survivors and those who died from MCC. Interestingly, several genes were linked to MCC-specific survival. Genes upregulated in deceased patients were primarily associated with angiogenesis and the PI3K-Akt and MAPK pathways, largely independently of viral status. In contrast, genes upregulated in survivors were linked to antigen presentation and immune response [53].

Moreover, inhibition of AKT resulted in the inactivation of mTOR and glycogen synthase kinase 3 pathway proteins while enhancing proapoptotic signaling by upregulating p16 expression and modulating the phosphorylation of the B-cell lymphoma-2-associated death promoter. The modulation of this signaling led to a strong and sustained suppression of virus-positive MCC cell proliferation, highlighting the potential clinical relevance of AKT inhibition for future MCC treatment strategies [54]. A further indication of the importance of PI3K/AKT/mTOR Pathway is that MLN0128, a dual mTORC1/mTORC2 inhibitor, effectively suppressed MCC cell growth both in vitro and in vivo using mouse xenograft models [55].

On the other hand, in a recent retrospective cross-sectional study on MCC alterations and tumor mutation burden (TMB), only a small subset of patients exhibited potentially actionable alterations [13]. It was determined that oncogenic alterations accounted for 20.2% of all detected variants. A general finding of the Brazel et al. study was that actionable alterations were more frequent in high TMB cases than in low TMB cases. Common level 3B variants included PIK3CA, BRCA1/2, ATM, HRAS, and TSC1/2, while frequent level 4 variants involved PTEN, ARID1A, NF1, and CDKN2A. Furthermore, the frequently affected pathways included RTK-RAS, TP53, cell cycle, PI3K, and NOTCH.

### 3.2. MAPK/ERK Pathway

The MAPK/ERK pathway is a signaling cascade composed of a series of intracellular proteins. Its activation begins at the cell membrane. This pathway plays a vital role in regulating key cellular processes, including proliferation, differentiation, survival, and apoptosis. It facilitates communication between signals received at the cell surface and the DNA in the nucleus, ultimately influencing gene expression [85,86,87,88,89].

The dysregulation of MAPK/ERK signaling has been observed in MCC, suggesting its role in tumor development [53]. Indeed, Sundqvist et al. showed that a subset of genes upregulated in deceased MCC patients was linked to MAPK pathway activation. This study found that their expression was largely independent of viral status in MCC patients [53]. On the other hand, the activation of JAK2 and MEK-ERK pathways was found to be more common in MCPyV-negative MCC compared to MCPyV-positive MCC. Additionally, the JAK inhibitor ruxolitinib effectively suppresses MEK-ERK pathway activation. Therefore, targeting the JAK-STAT and MEK-ERK signaling pathways may represent a promising therapeutic strategy for MCPyV-negative MCC [52]. Krasagakis et al. investigated the functional role of the KIT receptor and its ligand stem cell factor (SCF) in the pathogenesis of MCC. The activation of the KIT receptor by autocrine and paracrine exogenous soluble SCF enhanced the growth of MCC-1 cells, while it also upregulated the activation of the AKT and ERK 1/2 signaling pathway [90].

A gene expression profiling study highlights the importance of MAPK pathways in MCC, identifying two distinct and consistent molecular subtypes of MCC. Subtype I was linked to spliceosome function, DNA replication, and cellular pathways, while Subtype II exhibited the overexpression of genes involved in the TNF signaling and MAPK signaling pathways [56]. Recent studies suggest that MCPyV small tumor antigen (ST) expression contributes to the highly metastatic nature of MCC by enhancing cell motility and migration. This occurs through the differential regulation of cellular proteins involved in microtubule destabilization, filopodia formation, and the disruption of cell–cell junctions. However, the precise molecular mechanisms underlying these processes remain incompletely understood. Dobson et al., 2020 [91] show that MCPyV small tumor antigen (ST) expression activates p38 MAPK signaling, driving cell migration and motility. Specifically, it was demonstrated that p38 MAPK activation occurs independently of extracellular stimuli and is mediated through a non-canonical MKK4 signaling pathway. Notably, an interaction of the PI3K/mTOR and MAPK/ERK pathways is indicated when the PI3K/AKT inhibitor MLN0128 was combined with the MEK1/2 inhibitor trametinib. This combination exhibited synergistic effects in MCC cell lines, significantly altering the protein levels of downstream pathway components [57].

### 3.3. Notch Signaling Pathway

The Notch signaling pathway is a highly conserved cell–cell communication system that controls cell-fate determination and maintains homeostasis but also regulates crucial biological processes, including differentiation, proliferation, and apoptosis. It is activated when Notch receptors (Notch1-4) on the cell surface interact with ligands (Jagged1, Jagged2, Delta-like 1, 3, and 4) on adjacent cells. This interaction triggers proteolytic cleavage by γ-secretase, releasing the Notch intracellular domain (NICD). The NICD then translocates to the nucleus, where it binds to the CSL transcription factor (CBF1/RBPJ in humans) and recruits coactivators, leading to the transcription of target genes such as Hes and Hey family genes [92].

Notch signaling can operate through canonical and non-canonical pathways, where canonical Notch signaling involves NICD-mediated gene transcription, influencing cell fate decisions and tissue homeostasis. On the other hand, non-canonical Notch signaling is independent of CSL-mediated transcription. It interacts with different pathways, such as Wnt, PI3K/Akt, and NF-κB, contributing to various cellular functions [93]. Dysregulation of the Notch signaling pathway either promotes carcinogenesis or acts as a tumor suppressor, depending on the cell type [71,94].

Notch signaling, which influences cell fate determination and differentiation, has been implicated in MCC, affecting both the tumor cell and the MCC tumor microenvironment. Unlike other neuroendocrine malignancies, in a study that included 31 cases of MCC, NOTCH1 cytoplasmic and membrane expression was detected in 30 of them [58]. Alterations in Notch pathway components, including mutations and differential expression patterns, suggest a role in MCC tumorigenesis. Thus, a decrease in Notch expression was correlated to the increased necrosis and apoptosis of MCPyV-negative tumor cells, affecting their viability, growth, and migration [60]. These findings correlate well with another study showing that a comparison of gene expression and gene set enrichment analysis between 35 MCPyV-negative and 76 MCPyV-positive MCCs revealed significantly higher expression of EMT-related genes in MCPyV-negative cases. These genes were enriched in Notch signaling, TGF-β signaling, Hedgehog signaling, and the UV response pathway [61]. Another study, however, indicates that the expression of NOTCH1 and NOTCH2 showed no correlation with MCPyV status or prognosis. Furthermore, the expression of the Notch ligand, JAG1 expression, was significantly higher in MCPyV-negative MCC compared to MCPyV-positive MCC, while NOTCH3 expression was elevated in MCPyV-positive MCC. This study associated the expression of Notch3 with improved overall survival [59].

As a tumor suppressor, NOTCH3 expression is an independent prognostic marker for MCC outcomes. Therefore, even though Notch signaling is strongly associated with MCC pathogenesis, the exact impact of Notch signaling on MCC requires further investigation [12].

Notably, impairments of the Notch signaling pathway are associated with epidermal abnormalities, derangement of the surrounding epidermis, and cutaneous diseases such as atopy, inflammation, and cancer [95].

### 3.4. TP53 and RB Tumor Suppressor Pathways

The TP53 and RB1 genes encode the tumor suppressor proteins p53 and retinoblastoma protein (Rb), which are pivotal in regulating cell cycle progression and maintaining genomic integrity [96]. The P53 and RB tumor-suppressor pathways regulate DNA repair, cell cycle progression, cell death, and senescence, thus preventing the propagation of abnormal cells. They are commonly inactivated in several malignancies. Loss of function of these pathways occurs due to spontaneous alterations or even viral oncoproteins [97].

Retinoblastoma protein (RB) usually acts as a transcriptional coreceptor, prevents cell proliferation, and exerts a central regulatory effect on the cell cycle by controlling the transition from the G1 to the S phase. In its active hypophosphorylated state, Rb binds to E2F transcription factors, inhibiting the transcription of genes essential for S phase entry. Phosphorylation of Rb releases E2F, allowing cell cycle progression. Loss or inactivation of RB1 disrupts this control, contributing to tumorigenesis [96]. The interplay between the p53 and Rb pathways is crucial for tumor suppression. For instance, the E2F transcription factor, regulated by Rb, can activate the p53 pathway upon Rb dysfunction, leading to cellular senescence or apoptosis. This crosstalk ensures a robust defense against uncontrolled cell proliferation [97,98].

Inactivation of tumor suppressor pathways involving p53 and RB protein is common in MCC, particularly in MCPyV-negative tumors. Mutations in TP53 and RB1 genes lead to loss of cell cycle control, contributing to uncontrolled tumor cell proliferation. Regarding MCPyV-positive MCCs, pRB is targeted by the Large T antigen (LT) of MCPyV as research has shown that MCPyV-LT strongly binds to RB1 only, inactivating it and thus promoting MCC tumor growth in MCPyV-positive MCCs [68]. Interestingly, the prevalence of the copy loss of the RB1 gene among MCPyV-negative MCCs reaches over 60%, indicating that LT-negative MCCs maintain their pRB-negative characteristic [69]. In a study including 13 MCPyV-positive samples and 13 MCPyV-negative samples, RB expression was significantly higher in MCPyV-positive tumors and was associated with a superior overall prognosis [70]. In a study comparing the genomic copy number changes between MCPyV-positive and MCPyV-negative tumors, loss of the RB1 locus was primarily present in MCPyV-negative MCCs. At the same time, the RB1 promoter was hypermethylated in all MCC types regardless of their RB expression, setting this as a possible factor assisting the RB1 gene copy loss [70].

Inactivation of the p53 pathway has a known key role in tumorigenesis. In MCC, in addition to pRB dysregulation, p53 is also commonly disrupted. Specifically, MCPyV-negative MCCs present either inactivating mutations or deletions of TP53, whereas, in MCPyV-positive MCCs, their activity is downregulated [12]. Even though the pathogenesis of MCC is still under investigation, one of its leading causes is thought to be UV exposure. UVB-specific mutations have been identified in the p53 tumor suppressor gene, setting this as a main pathway in the pathogenesis of this malignancy [62,63].

Unlike PRB, research demonstrated that in MCPyV-positive MCCs, LT is not proven to bind to p53 [99], hence reinforcing the hypothesis that either MCPyV Small T antigen (ST) or structural remodeling of the genome of the MCC cell caused by MCPyV itself could affect the activity of p53. This is corroborated by a study that included tumors from 91 patients, which demonstrated that TP53 mutations were only present in MCPyV-DNA-negative tumors, highlighting another distinct feature between the two MCC subtypes [64].

Further studies have revealed a complicated dual-targeted controlling system of p53 managed by MCPyV T antigens. The binding of MCPyV-LT to pRB results in the accumulation of p14, a cyclin-dependent kinase inhibitor (normally inhibiting MDM2), which activates the p53 signaling pathway. At the same time, MCPyV-ST downregulates p53 by increasing the levels of MDM2 and CKα, which cooperate with MDM4 [65].

Although crucial in the pathogenesis of MCC, P53 inactivating mutations are relatively low in MCC and studies have demonstrated that their prevalence floats around 10%. Lassacher et al. showed that p53 mutations are present in 14% in a study that included MCCs from 21 elderly patients [66]. Consistent with this, in a study that included MCC cell lines, p53 mutations were similarly present in 14% of them, while in the remaining cell lines, wild-type sequences in the p53 mutation hot spot region were demonstrated [67]. In the same context, Houben et al. investigated the effect of tumor antigens (TAs) on p53 expression. Interestingly, overexpression of TAs did not restrain p53 expression and at the same time, TA knockdown demonstrated no effect on p53 and p53 target genes expression, while HDM-2 antagonism exhibits promising effects on the activity of p53. This has resulted in the discontinuation of clinical studies [67].

### 3.5. Wnt/β-Catenin Pathway

The Wnt/β-catenin pathway was discovered in 1982 and has an established role in fundamental physiological cellular processes such as proliferation, differentiation, migration, genetic stability, apoptosis, and stem cell renewal [100]. Its dysregulation is pivotal in the carcinogenesis and progression of various malignancies due to uncontrolled cell growth.

**Figure 2:**
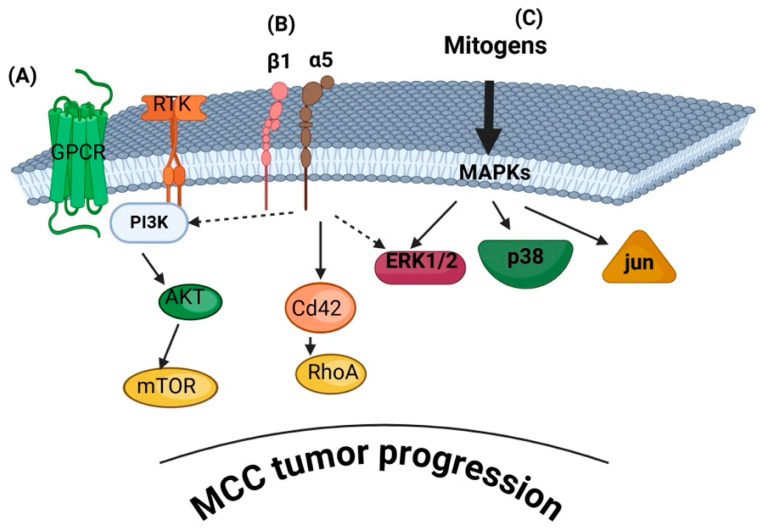
Signaling mechanisms in MCC tumor progression. (**A**) The PI3K/AKT/mTOR signaling axis is triggered by upstream RTK and GPRC; (**B**) Integrin downstream signaling including Cd42/RhoA with cross-reaction with PI3K and ERK1/2. (**C**) Mitogen signaling via MAPKs/ERK1/2 and jun. Created in BioRender. Nikitovic, D. (2025) https://BioRender.com/g86i995 (accessed on 28 February 2025).

The Wnt/β-catenin signaling pathway, known for its role in cell proliferation and differentiation, has been investigated in MCC. Exposure to UV radiation as well as advanced age, which are known risk factors for MCC, trigger the Wnt/β-catenin pathway, stimulate MMPs, and may facilitate MCPyV infection [38]. However, studies have demonstrated that the accumulation of β-catenin is infrequent in MCC, suggesting that this pathway is not a primary driver of MCC pathogenesis [71,72]. A summary of signaling pathways involved in MCC development and progression is summarized in Figure 2.

### 3.6. Extracellular Matrix (ECM)-Derived Signaling in MCC

#### 3.6.1. Integrin Signaling

The ECM is a critical component of the tumor microenvironment, influencing cancer cell behavior through biochemical and mechanical cues. Integrins are transmembrane receptors mediating cell-m ECM interactions. Activation of integrin pathways can lead to downstream signaling cascades promoting tumor progression [101]. Upon binding to ECM components, integrins undergo conformational changes that initiate intracellular signaling cascades, notably activating pathways such as PI3K-AKT and NF-κB, which promote cell survival and proliferation. Additionally, integrin engagement can lead to increased expression of anti-apoptotic proteins like BCL-2 and FLIP, further enhancing tumor cell survival. Integrins also regulate the dynamics of focal adhesions and the actin cytoskeleton, facilitating cancer cell migration and invasion. Moreover, integrin signaling contributes to activating matrix metalloproteinases (MMPs), enzymes that degrade the ECM, thereby promoting tumor metastasis. Given their central role in these processes, integrins are considered promising targets for therapeutic intervention in cancer treatment [102,103,104].

In MCC, cell adhesion, migration, and survival promote its aggressiveness. Such cancer cell functions depend on the action of integrin receptors [104]. Furthermore, integrins were identified in MCC cell line-derived exosomes [38], suggesting their action in MCC function. Activation of integrin pathways can lead to downstream signaling cascades, including the PI3K/AKT and MAPK pathways, promoting tumor progression [105]. In the MCPyV-positive MCC cases, expression of the small tumor antigen (ST) has been suggested to modulate cell motility. A recent study identified an integrin β1 involvement in MCC cell filopodia formation. Thus, MCPyV ST was found to alter the actin cytoskeleton, driving filopodia formation, a key process for MCPyV ST-induced cell motility. Additionally, the involvement of specific Rho family GTPases, Cdc42 and RhoA, was determined in these mechanisms. Furthermore, a novel pathway for Rho-GTPase activation and cell motility regulation was shown, mediated by the interaction between MCPyV ST and the phosphatase catalytic subunit PP4C, which results in the targeted dephosphorylation of one or more integrins (mainly integrin β1) [35]. The role of integrins in MCC tumors was also investigated by Akgul et al. using different MCC cell lines [73] based on the characteristic that MCPyV-negative cell lines [106,107,108,109] are mostly adherent cell lines compared to MCPyV-positive ones [108,110] that grow in suspension [73].

Expression of different α and β integrin isoforms were analyzed and presented that β5 integrin is highly expressed, both in the mRNA and protein level, in adherent (mainly MCPyV-negative) cell lines compared to cell lines grown in suspension (MCPyV-positive), where only traces of β5 integrin were detected [73]. In addition, immunohistochemical analysis on tissue sections of MCC cases concurred with the viral positivity being inversely correlated to the lower expression of β5 integrin [73]. Therefore, MCPyV was shown to affect integrin expression, which could lead to differences in the adhesion, proliferation, and migration capacities of Merkel cells.

Extracellular vesicles, particularly exosomes, serve as an evolutionarily conserved mechanism for intercellular communication. Their role in various pathologies, including cancer, has garnered significant attention, highlighting their potential as prognostic and diagnostic biomarkers and as therapeutic tools. However, a comprehensive secretome analysis for MCC remains unexplored. To investigate the possible contribution of exosomes in MCC, Konstantinell et al. analyzed the protein content of MCC-derived exosomes [36]. Since approximately 80% of MCC cases are MCPyV-positive, secretomes of two MCPyV-negative and two MCPyV-positive MCC cell lines were compared. These authors identified 164 exosome-associated proteins common to all four cell lines, confidently annotated in the ExoCarta and Vesiclepedia databases. These proteins include key cell motility, metastasis, and tumor progression regulators, such as integrins and tetraspanins, as well as intracellular signaling molecules, chaperones, proteasomal proteins, and translation factors [36]. These data indicate a likely important role of integrins, which remains to be fully elucidated.

#### 3.6.2. ECM Remodeling Enzymes

MMPs and other ECM-modifying enzymes alter the ECM composition and stiffness, affecting cell signaling. The role of MMPs in tumor invasion and metastasis has been extensively investigated [111]. Increased MMP activity in MCC can enhance tumor invasiveness by degrading ECM components and releasing bioactive molecules that promote angiogenesis and metastasis [43]. Thus, changes in MMP expression also aid the virus-enhanced cancer development. For example, MCC tumor cells expressing T antigens were found to produce higher levels of MMPs [112].

Tissue homeostasis is sustained through the action of enzymes involved in the synthesis and degradation of the ECM. Abnormal ECM degradation can lead to pathogenesis like tumor formation [113]. Matrix metalloproteinases (MMPs), a zinc-dependent family of proteolytic enzymes, are well-known modulators of ECM degradation and remodeling [101,114]. MMP-9, an MMP isoform known to regulate cancer cell function, is enhanced in MCPyV-positive MCCs [115]. The MCPyV-ST, a key oncoprotein driving MCPyV-positive MCCs, is known to modulate motility in MCC cells and enhance MMP-9. Nwogu et al., 2020, demonstrated that MCPyV-ST also induces the expression of EMT-associated genes, including MMP-9 and Snail, in a process dependent on the large T stabilization domain (LSD), which disrupts FBW7, a key regulator of MMP-9 and Snail. Inhibiting MMP-9 significantly reduced MCPyV-ST-driven cell migration and invasion, highlighting MMP-9 as a potential therapeutic target in MCC [74]. Interestingly, MMPs secreted by the dermal fibroblast were recently implicated in participating in MCPyV infection. Liu et al., created an in vitro ex vivo model of dermal cells representative of the dermal compartment. Using this model, Liu et al., 2016, found that the WNT/β-catenin signaling pathway and other growth factors induce MMP1, MMP3, MMP7, MMP9, MMP10, MMP11, and MMP13 gene expression, which in turn enhances MCPyV infection. This suggests that MCC risk factors, such as UV radiation and aging, which are known to activate WNT signaling and MMP expression, may facilitate viral infection and contribute to MCC development [38].

Earlier studies using immunohistochemistry on tissue sections of surgically resected MCCs showed a significant correlation between the metastatic capacity of the tumor tested and high expression levels of MMP 7 and MMP10/2 [40]. In the same study, there was also found a statistically significant correlation between the expression of MMP7—VEGF, MMP7—P21, MMP7—P38, and MMP10/2—VEGF [40]. Furthermore, immunohistochemical analysis of 44 primary MCC tumors and 6 lymph node metastases revealed distinct expression patterns of MMP-10, MMP-21, MMP-26, and MMP-28. MMP-28 was found in tumor cells, particularly in smaller tumors (<2 cm), and in tumor stroma, MMP-21 was detected in tumor cells. In comparison, MMP-26 was expressed in stromal cells and was associated with larger tumors (≥2 cm) and poor prognosis. MMP-10 was the most frequently expressed and was prominent in metastatic lymph nodes. In UISO MCC cells, MMP-10, MMP-21, and MMP-28 mRNAs were basally expressed, with IFN-α and TNF-α downregulating MMP-21 and MMP-28. These findings suggest that MMP-26 is linked to aggressive MCC, while MMP-21 and MMP-28 may be associated with tumors of lower malignant potential [39]. These findings demonstrate the importance of ECM reorganization signals in the pathogenesis of MCC.

#### 3.6.3. Mechanotransduction

Changes in ECM during cancer development affect the mechanotransduction pathways and properties in cancer cells, like MCC cells. The mechanical properties modulated include stiffness, fibrillar collagen orientation, and cross-linking density [30]. In addition, mechanosensing signaling molecules such as focal adhesion kinase (FAK), Yes-associated protein (YAP), cadherins, integrins, and syndecans are activated and influence cell proliferation, migration, and metastasis [30,116]. Although aberrant activation of YAP1 (Yes1-associated transcriptional regulator) has been reported for several solid cancers, in MCC specifically, an inverse correlation between a neuroendocrine gene signature and the Hippo pathway transcription factor YAP1 was identified at the transcript and protein level in MCC tumor biopsies as well as in established and patient-derived cell lines, distinguishing MCCs based on their intratumoral heterogeneity [117]. The tumor microenvironment also includes the behavior of cancer-associated fibroblasts (CAFs), endothelial cells, and immune cells that can influence metastatic potential [30]. CAFs have been characterized as desmoplastic cells. Their activation increases various growth factors, cytokines, and chemokines, which aid cancer development and angiogenesis.

Albertini et al. showed that CAFs can differentially enhance MCC growth and angiogenesis [21]. The same study also presented the role of the renin-angiotensin molecular pathway and, more specifically, the aminopeptidase A/angiotensin II and III/angiotensin II type 1 receptor (AT1R) axis in affecting MCC development [21]. Furthermore, investigation of the expression of Piezo channels, mechano-gated ion channels essential for mechanotransduction in mammalian cells, in a case series of Merkel cell carcinoma detected PIEZO2 expression in the membrane of all cells and the perinuclear area [118].

To date, the mechanical regulation of MCC by the tumor microenvironment is still unknown, and future studies are crucial in describing the mechanosensing and signaling pathways involved in MCC development. Figure 2 depicts the key signaling pathways implicated in MCC pathogenesis.

## 4. Immune Traits in Merkel Cell Carcinoma

In most cases, MCC is an immunogenic tumor driven by Merkel cell polyomavirus (MCPyV) oncoproteins. MCPyV-specific T-cell responses influence the clinical outcome of patients diagnosed with MCC. As MCPyV oncoproteins are immunogenic, they force tumor cells to develop intense immune evasion mechanisms. These evasion mechanisms rely on the downregulation of MHC expression, up-regulation of PD-L1 as inhibitory molecules, and the intense secretion of immunosuppressive cytokines/chemokines. This section will present arguments for various immune traits of MCC [119].

### 4.1. Immune Characteristics of Tumor Tissue

The immune pattern of MCC is governed by two major players: the tumor cell and the presence of the tumorigenic MCPyV infection.

Tumor-infiltrating lymphocytes (TILs) are one crucial parameter when evaluating the pathology of solid tumors, depicting the possible in situ anti-tumor response. The immune pattern of the cancer is not included in the AJCC staging for MCC. However, an extensive analysis of databases reported in 2020 by Yusuf et al. has shown that TIL can aid clinical prognostic factors in MCC. Therefore, histopathologic TIL grade could independently predict the patients’ overall survival (OS) and more detailed TIL immunophenotyping could increase its predictability in the patient clinical outcome [120]. In MCC, TILs consist mainly of the CD4+, CD8+, and regulatory T-cell (Treg) subpopulations, with these cells sustaining the immune responses against a developing tumor. In MCC, there are also MCPyV-specific immune cells as a response to the viral infection [16], and in MCPyV-positive tumors, a higher CD8+ T infiltration was reported [121].

Using multiplex-IHC/immunofluorescence, within TILs, new T subpopulations were identified consisting of a high percentage of CD4/CD8 double-negative (DN) T-cells. These T cells are closely linked to inflammatory conditions in various diseases, including cancer. DN T cells were predominantly Vδ2-γδ T cells that prove their anti-tumor action and express PD-1 and lymphocyte activation gene 3 (LAG3); their phenotype describes an immune exhausted sub-population. Moreover, as the γδ T-cell exhibit anti-tumoral actions, their infiltration can be a prognostic biomarker in MCC [122]. A study published in 2024 evaluated several immune markers in non-treated patients. The study confirmed that LAG-3 is highly expressed in MCC infiltrates and is probably a prognosticator of MCC. Moreover, as confirmed by earlier studies, CD8+ and γδ T densities can be novel biomarkers in MCC [123].

Moreover, Donizy et al. reported that PD-L1 expression, increased intratumoral CD8+T cells, and FoxP+ lymphocytes could be favorable prognosticators in MCC. This CD8+ FoxP+ T cell subpopulation does not have immune suppressive potency like its Treg CD4+ FoxP+ subpopulation but instead develops characteristics of tissue-resident memory and effector T cells [124].

As MCC has approved immune therapies aiming at PD-1/PD-L1 as further detailed, intense research has been performed on the prognosticator capacity of the expression of these two molecules. Within TILs, PD-1 and PD-L1 have different expressions; while PD-1 was found to be expressed on over 90% of the investigated tumors, a study published in 2021 did not find PD-L1 expression on any of the tumor cells [121].

Tumor regression is understudied and poses an essential subject for many groups of researchers. The phenomenon rarely appears spontaneously but is induced by the treatment. Spontaneous regression of MCC is a rare event, but a study published in 2021 showed that the regressed tumors were 75% MCPyV positive. In these cases, CD3+ T cells were found in the peritumoral zone [125].

As interferons (IFN) are key players in tumorigenesis, in MCC cell lines, Saurer et al. have shown the transcriptomic pattern of these cells subjected to IFNs. An upregulation of the genes involved in the immune escape was detected upon IFNγ in vitro treatment [126]. In vitro IFN-γ treatment of patient-derived MCC cell lines revealed remarkable upregulation of class I gene transcripts. HLA I is expressed on the cell membrane of most cells and its loss serves as a known widespread mechanism of immune evasion in cancer. Reversal of this loss or HLA-I upregulation may further enhance immune therapy in MCC [127]. More recently, in 2024, single-cell RNA sequencing (RNA-seq) of MCC tumors has proven, besides increased tumor proliferation and neuronal stem cell markers, the active transcription of IL-1. Patients that responded positively to immune therapy had increased type I/II IFNs and resident CD8+ or Vδ1 γδ T cells within their tumor tissue. Moreover, through investigating spatial transcriptomics within the tumors, colocalization of T and B lymphocytes with dendritic cells was shown. This colocalization creates favorable grounds for secreting stimulatory cytokines and chemokines [128].

Using RNA-seq technology, the genes associated with MCC-specific survival were found along with up-regulated genes correlated with worse clinical outcomes of MCC patients. In this later group, upregulated genes were associated with angiogenesis and intracellular pathways (PI3K-Akt and MAPK). Genes found upregulated in good outcomes in MCC patients were associated with antigen presentation and immune response [53]. 

Cancer-associated fibroblasts (CAFs) promote tumorigenic milieu by initiating the remodeling of the extracellular matrix and secreting pro-tumoral cytokines/chemokines. CAFs were studied in MCC in association with TILs. As high-intra-tumoral CD8+TIL was also confirmed as associated with a good clinical prognosis, a negative association was reported between TILs and CAF expressing high levels of IL-6. IL6+CAFs probably modulate the tumor immune microenvironment and the activity of T-lymphocytes [22].

### 4.2. Circulatory Immune Parameters

Besides the immune pattern of the actual MCC tumor, recent studies have also evaluated the circulatory and immune parameters of MCC patients to find new prognosticators. In a study where both melanoma and MCC patients were evaluated, using flow cytometry and cell sorting, circulating CD8+ T-cells that express PD-1+TIGIT+ were found in patients after 1 month of anti-PD-1 therapy. The existence of this circulatory population was associated with a good clinical response and represented tumor-specific T-cells, overexpressing CXCR5 (a marker for CD8+ cytotoxic follicular T-cell lymphocytes). T cell immunoreceptors with immunoglobulin and ITIM domain (TIGIT) and PD-1 have a synergic action as immune checkpoints when PD-1 signaling is impaired [129].

### 4.3. Immune Patterns Induced by MCPyV

Besides the tumor cell, the other immune pattern studied in MCC is induced by the MCPyV infection. This infection is present in up to 80% of the cases [130] linked to the expression of MCPyV T-antigen (T-Ag). Jing et al. have shown that when TILs are stimulated with an artificial antigen-presenting cell (aAPC), 75% of the tested MCC patients had CD8+T cells recognizing MCPyV epitopes. Interestingly, almost all patients with MCPyV+ tumors showed HLA alleles that can restrict CD8+ T-cell responses to MCPyV T-Ag, and the oncogenic domains of the antigen were not commonly recognized [131]. Schlemeyer et al. have shown that viral oncoprotein small T antigen (ST) specifically hinders selectin ligand binding and further antigen processing of MCC’s surface molecules. This immune hindrance alters tumor cell immune recognition. Moreover, sT, through CD47 regulation, inhibits the phagocytosis process performed by macrophages.

Biological serum samples from over 200 aged non-MCC subjects were tested for anti-MCPyV IgGs, and over 60% were found positive. These data published in 2021 suggest that in elderly subjects, this oncogenic MCPyV circulates, and if immune suppression is installed, this virus can further drive MCC carcinogenesis [132]. A similar study published in 2023 by the same group performed on various age individuals has shown that in children (0–5 years), the lowest anti-MCPyV IgG was found and that primary infection with MCPyV (e.g., appearance of IgM) occurs during early childhood. IgG peaks at about 6–10 years old and remains stable throughout life, inducing long-lasting antigen stimulation [133].

**Figure 3:**
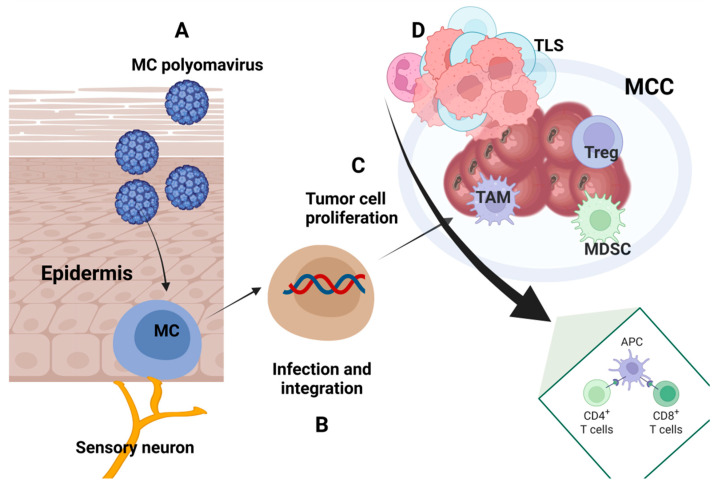
Main immune processes developed with MCC. (**A**) Epidermis subjected to various extrinsic factors, like UV irradiation or polyomavirus infection, can suffer various alterations; (**B**) Merkel cell (MC) in close connection with the sensory neurons within the dermis can be subjected to infection and integration of viral genetic material; (**C**) If additional factors contribute, e.g., aging, the infected MC can lead to tumorigenesis, developing a Merkel cell carcinoma (MCC); (**D**) Within the tumor, immune suppressive cells (tumor-associated macrophages TAM, myeloid-derived suppressor cell MDSC, regulatory T lymphocytes Treg) can favor tumor progression. Tumor antigen presentation is sustained by antigen-presenting cells (APCs) to prime T cells (can be performed in the lymph nodes or as depicted here in the tertiary lymphoid structures (TLSs) at the peritumoral site.

The MCPyV replication induces an inflammatory cytokine and IFN-stimulated gene (ISG) activation. Changes induced in the skin microenvironment through natural aging or immunodeficiency can lead this viral infection to induce MCC’s carcinogenesis [134]. sT antigen regulates the type I IFN by interfering with the interferon-stimulated gene factor 3 (ISGF3)-ISG. These early viral proteins trigger the type I IFN response, influencing MCPyV infection and regulating the MCC tumor microenvironment [135]. It was shown that MCPyV infection could be induced in the MCC tumor CD3, CD8, FoxP3, and PD-L1 positive cells [136], confirming the fact that the viral infection increases the tumor’s immunogenicity [137]. A general outline of the immune processes in the MCC is presented in Figure 3.

## 5. Histopathology of MCC

**Figure 4:**
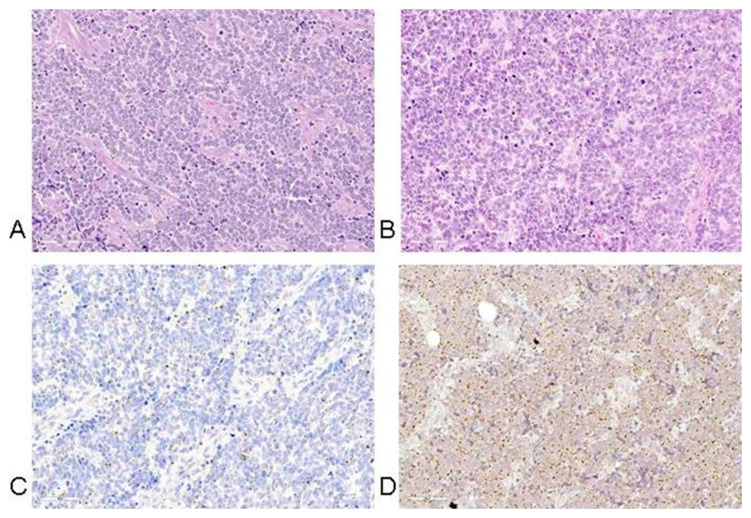
Merkel cell carcinoma histopathology and immunohistochemistry. (**A**,**B**) Sheets, trabeculae, and cords of small cells with large nuclei and small amounts of cytoplasm; nuclei with fine granular/clear chromatin; indistinct nucleoli; and numerous mitoses. HE ×400; (**C**,**D**) Perinuclear positivity (dot-like) for CK20 (**C**) and AE1AE3 (**D**). (**C**)—CK20 ×400; (**D**)—AE1AE3 ×400.

Histopathological studies of MCCs are crucial in diagnosing and differentiating this tumor from other malignancies. Standard hematoxylin-eosin staining of skin MCC biopsies typically consists of small monomorphic round blue cells with scant cytoplasm, indistinct nucleoli, abundant mitoses, and dense cytoplasmic granules. The cells most frequently form nodules, with a characteristic infiltration of small cells arranged in sheets, nests, and rarely ribbons separated by abundant stroma (Figure 4) [138,139,140]. Further histopathological studies of MCCs have shown the proliferation of small basophilic tumoral cells in the dermis and occasionally the hypodermis, with the majority of MCC tumors extending to the subcutaneous tissue rather than remaining confined to the dermis [141,142]. Epidermotropism and epidermal ulceration are present in less than 10% of the cases studied, while a space dividing the tumor and the epidermis is more commonly present [143,144,145]. Mitotic figures and necrotic cells are also common histological features of MCC, while vascular invasion and profuse inflammatory infiltration of lymphocytes and plasma cells surrounding the tumor are also commonly present [145]. 

In MCC, mitoses are frequent and the apoptosis index is high. The histologic features, tumor thickness, microanatomic compartment involved by tumor (dermis and/or subcutis and/or deeper), tumor growth pattern (nodular circumscribed versus infiltrative), lymphovascular invasion, tumor-infiltrating lymphocytes, tumor necrosis, ulceration, and solar elastosis, are parameters that should be reported with regard to prognosis. Overall, tumor thickness, the presence of a nodular growth pattern, low tumor depth, and absence of lymphovascular invasion were statistically significantly associated with longer survival of MCC patients [142].

Albertini et al. studied the functional role of cancer-associated fibroblasts (CAFs), which are the principal elements of the tumor microenvironment, via subcutaneous coinjection with MKL-1 cells into immunocompromised mice. Interestingly, the patient-derived CAFs seemed to promote tumor progression and metastasis. Human CAFs were found mainly around blood vessels, and there is evidence that they exert proangiogenic activity in MCC [21].

Basic characteristics of MCC tumor histopathology are presented in Figure 4.

Morphological studies described that MCPyV-negative MCCs more frequently presented elongated nuclei compared to MCPyV-positive MCCs [146]. MCPyV-negative MCCs also displayed a neuroendocrine carcinoma phenotype with larger cell size, abundant cytoplasm, and prominent nucleoli [146]. In addition, expression experiments demonstrated that MCPyV-negative MCCs had higher expression levels of thyroid transcription factor 1, cytokeratin 7, glypican 3, no or high levels of p53, and no neurofilament expression [146,147]. MCPyV-positive MCCs expressed cytokeratin 8, 18, 20, CD99, EMA as well as the transcription factors c-myc and LEF1 [146,148]. In addition, the CD99 expression had a dot-like expression pattern that was highly related to MCPyV [146]. Ricci et al. studied the immune checkpoint receptor CD279/PD-1/PDCD1(mPDCD1) expression, which is related to clinico-pathological features of MCCs, showing that mPDCD1 is present in higher levels in MCPyV-positive MCCs and is related to >75 years of age, absence of immune cells, and no PD-L1 expression [149]. In contrast, a study investigating the expression of the methylation of the human telomerase reverse transcriptase (mhTERT) showed no difference between MCPyV-positive and -negative MCCs [150]. Furthermore, both positive and negative virus MMCs highly expressed oncogenic transcription factors such as the nuclear factor of activated T cells (NFAT), p-CREB, and p-STAT3, demonstrating shared pathogenic mechanisms [148].

Divergent differentiation of MCC has been associated with higher aggressiveness and MCPyV negativity, suggesting that MCPyV might not be involved in the carcinogenesis of this tumor subtype. Interestingly, various divergent components have been identified among the typical histological features of MCC across multiple case studies. Occasional cases have shown focal areas exhibiting squamous, melanocytic, glandular, muscular sarcomatous (fibrosarcomatous, leiomyosarcomatous, rhabdomyosarcomatous), or lymphoepithelioma-like features of differentiation, while aberrant divergent components have also been determined in fewer cases [145,151,152,153,154,155,156,157,158].

Unique immunohistological changes have been detected in MCC cases that exhibit spontaneous regression. Although rare, complete spontaneous regression has been linked with improved prognosis and associated with MCPyV positivity [159]. The exact mechanism remains debated, but other than peritumoral fibrosis, infiltration of CD4+ and mainly CD8+ was seen in the tumor nests following the biopsy of an MCC case, reinforcing the hypothesis of biopsy-induced T-cell immune stimulation [160].

Positive and differential diagnosis is based on immunohistochemical tests. Most cytokeratins (except CK7) are positive in tumor cells with a characteristic paranuclear dot-like appearance; CK7 is positive in rare cases. Positivity for CK20 is specific for MCC, with almost 97% of tumors being positive and showing a paranuclear dot-like pattern. Concomitant positivity for neuroendocrine markers is recorded (CD56—membranous expression, chromogranin, and synaptophysin—cytoplasmic staining and INSM1—nuclear positivity) [161]. Neurofilament (NFP) is also positive and useful in rare CK20-negative MCCs. MCC negativity for melanocytic markers, lymphoid markers, and TTF1 is helpful in differential diagnosis with other small round blue cell tumors [162].

Although crucial, the histopathological diagnosis of MCC can be quite challenging due to its overlapping features with a variety of other tumors such as basal cell carcinoma, melanoma, Ewing sarcoma, neuroblastoma, leukemia cutis, and metastatic small cell carcinoma of the lung [138,163]. Specifically, the formation of clusters that may develop in the epidermis or their configuration into pagetoid patterns may mimic other intraepidermal malignancies such as melanoma, mycosis fungoides, Bowen disease, or Paget’s disease [164].

## 6. Therapy

Standard treatment options for patients with MCC include surgery, adjuvant radiotherapy, chemotherapy, and immunotherapy, which presently comprise mainly PD-1 and PD-L1 inhibitors [165].

Typically, surgery is the first-line treatment for biopsy-confirmed MCC in patients who do not have comorbidities that make them unsuitable surgical candidates. According to the recent NCCN 2024 guidelines, the tumor is resected along with a wide area of 1–2 cm of surrounding skin, considered the cancer-free margin [62]. Excision and sentinel lymph node biopsy (SLNB) is recommended, regardless of clinical evidence of sentinel lymph node infiltration, as it ensures crucial information regarding the staging of MCC and determines further therapeutic actions [62,166,167]. A further personalized management plan is chosen based on the AJCC TNM staging system [18]. In MCCs where distant disease is absent, surgery and adjuvant radiotherapy are recommended to ensure maximum locoregional control, while systemic treatment is suggested in the presence of distant metastasis confirmed with imaging studies [62]. A recent systematic review, including studies published between 1972 and 2023, revealed that adjuvant radiotherapy reduces the risk of death and improves disease-free survival among early-stage MCCs. Although surgery offers improved local control, adjuvant radiotherapy appears to be more beneficial compared to surgical monotherapy [168]. As chemotherapy exhibits limited efficacy, the emerging immunotherapies seem to be a promising tool for managing disseminated MCCs [55].

### 6.1. Immune Therapy in Merkel Cell Carcinoma

Immune therapy has changed the clinical scenario of a multitude of cancers. For MCC-diagnosed patients, there are currently three approved antibodies by the Food and Drug Administration (FDA): avelumab as anti-programmed death-ligand 1 (anti-PD-L1) and pembrolizumab and retifanlimab as anti-PD-1 monoclonal antibody treatments. A recent large database study including patients with MCC with clinically detected regional lymph node metastasis, highlights the improved 5-year overall survival of the group of patients who received neoadjuvant immunotherapy, compared to the group of patients who did not receive neoadjuvant immunotherapy, suggesting the significant contribution of immunotherapy in MCC [169]. Biomarkers for the efficacy of immune therapy are still to be established. As highlighted above, PD-L1 expression as a biomarker for efficacy still has conflicting results [170]. Tumor heterogeneity of the same tissue section can explain why PD-L1 expression did not qualify as a predictive biomarker for immune therapy in MCC [171].

Avelumab tested in an observational real-world study demonstrated its high response rate, having durable responses and prolonged survival [172], proving an OR of almost 60% and that it is effective even in immunocompromised subjects [173]. Multiplexed immunohistochemistry performed on tumors harvested from patients subjected to PD-1/PD-L1 therapy demonstrated that TILs containing CD8+ effector and central memory T cells (TCMs) were associated with a good clinical response [174]. A recent systematic review published in 2024 evaluating the PD-L1/PD1 blockade in MCC has confirmed that patients have durable responses and good survival outcomes [175].

There are multiple tentative to evaluate efficacy biomarkers in MCC patients. Amongst responders to immune therapy, TCM expression of genes that encode processes like lymphocyte attraction and activation was identified. At the same time, in TILs, low T-cell clonality but high TCR diversity was found. Conversely, in non-responders, a constrained TCR repertoire of T lymphocytes was identified [176]. Recent studies also reported innate immunity’s role in the responders to immune therapy patients. Single-cell transcriptomics on MCC tissues recognized tumor-associated macrophages (TAMs) as the main myeloid cells. TAMs express PD-L1 and the Leukocyte immunoglobulin-like receptor subfamily B member 1 (LILRB). High infiltration of TAMs (phenotype CD163+, CD14+, S100A8+) was associated with resistance to PD-1 therapy and hence cells that sustain immunosuppressive processes [177].

Regarding non-responders and the acquired resistance, a recently identified mechanism of PD-1 resistance was reported. The activation of protumorigenic mTOR signaling, mitochondrial respiration, and reactive oxygen species (ROS) generation was incriminated for the acquired resistance. This axis sustains a pro-tumorigenic milieu and accelerates tumor growth [178].

A recent study investigated somatic mutations, mutational patterns, and tumor mutational burden in MCC patients along with PD-L1 expression and CD8+T infiltration. MCPyV+ tumors displayed high percentages of immunosuppressive M2 macrophages, while high CD8+ T-cell percentages did not predict response to immune therapy with avelumab. In opposition, MCPyV- tumors, displaying higher CD8+ T-cell percentages, were associated with a good clinical response to avelumab [179]. The durability of the approved immune therapy was studied and reported by Ramadoss et al. Within almost 200 MCC-treated patients (over 75% treated with pembrozulimab, over 22% on avelumab, and 2% on nivolumab), nearly 50% had a complete response to initial immune therapy. In contrast, the other half of the patients experienced disease progression at a median of 11.3 months from therapy cessation. A higher risk for progression was observed in patients with MCPyV+ tumors compared with the negative ones. Therefore, virus-positive MCC can be a risk factor for post-discontinuation relapse [180,181]. Hence, further studies are needed to evaluate the optimal duration and the durability of response after cessation [181].

### 6.2. Other Immune-Mediated Therapies

Many studies have focused on MCC as the stimulator of interferon genes (STING). STING is essential to innate immunity, so it induces type I IFN [182]. In various cancers, MCC included, STING is found silenced; therefore, activating it can turn immunologically “cold” tumors into “hot” ones. Liu et al. have used mRNA-lipid nanoparticles to deliver active STING mutant (STINGR284S) to cancer cells. The delivery of STINGR284S reactivated the antitumor response without any side effects [183]. In experimental models, a human STING mutant (STINGS162A/G230I/Q266I) was delivered to MCC cells, reactivating antitumor cytokine/chemokine–based processes. Co-cultivating these MCC cells with T cells and MCPyV-specific TCRs increased anti-tumor activity. Thus, targeted delivery of STING in tumor cells can be a new therapy approach [184]. More recently, an intratumoral STING agonist (ADU-S100) combined with an anti-PD-1 antibody was tested in MCC patients. At baseline, 12% of T cells were MCPyV-specific T lymphocytes in TILs exhibiting exhaustion markers (e.g., high TOX and low TCF1 protein expressions). Post-treatment, CD8+ T cells actively expanded, and HLA-I expression increased in tumor cells. High STING expression was identified in immune and stromal cells, and these results showed that STING agonists interfere via signaling pathways in MCC through immune and stromal cells [185].

MCPyV infection of dermal fibroblasts (DFs) can induce type I and III IFNs, causing the activation of ISGs. Molecular mechanisms pinpoint that type I IFN directly affects MCPyV infection by repressing early viral transcription. Moreover, the study shows that UV-irradiation significantly stimulates MCPyV gene expression and replication. The observation that type I IFN can repress viral oncogene transcription MCC virus-positive can benefit from future therapies based on this cytokine family [186].

Other studies open new treatment avenues in MCC-like vaccines and Adoptive T cell transfer (ACT) cell treatments. Thus, Wong et al. reported in 2020 that in the peripheral blood of MCC patients, circulating CD8+ T cells reactive to MCPyV T antigen (T-Ag) were subjected to anti-PD-1 immunotherapy [187]. Over 80% of the MCC tumors express antigens of MCPyV that are ideal for TCR-based immunotherapy. Asano et al. reported in 2024 that the identification of TCRMCC1 recognizes a T-antigen epitope in HLA-A*02:01 restriction and demonstrates the possibility of using TCR gene therapy in MCC [188].

As MCPyV integrates into the patient’s genome, the oncoproteins expressed by tumor cells can be important targets for an eventual vaccine. Buchta et al. designed a vaccine that induces an antigen-specific CD4 T cell against MCPyV-LT (LTS220A-UNITE™ DNA vaccine) (ITI-3000). The report shows the results of pre-clinical studies in which ITI-3000 vaccination enhanced specific CD4 T cell responses, increased anti-tumor immune responses, and was the ground to initiate the clinical study in polyomavirus-positive MCC patients (NCT05422781-ITI-3000: Merkel Cell Carcinoma—Immunomic Therapeutics) [189]. A multiepitope vaccine against the virus was designed using integrated immunoinformatics and vaccinomics. MCPyV action has been related to the expression of small T and large T oncogenes. The histone methyltransferase enhancer of zeste homolog 2 (IFN2), an activator of the H3K27 trimethylation, has been suggested as a therapy agent in MCC [190,191]. Durand et al. demonstrated that the majority of MCC tissue sections studied expressed EZH2 (92%). EZH2 in MCC additionally had a higher expression in virus-positive compared to virus-negative MCC tissues [191]. Furthermore, large T antigen changes in expression, by fibroblasts, modulated EZH2 expression, resulting in the cytotoxicity of MCPyV-positive MCC cell lines. In addition, knockdown experiments showed that T antigen and EZH2 expression are crucial for MCPyV-positive MCC growth [191]. In a nutshell, the designed vaccine contains the highest antigenic, epitopes recognized by cytotoxic T lymphocytes, helper T lymphocytes, and B lymphocytes, linkers to immunogenicity, and binding sites for TLR4 to increase its adjuvancy [192]. Vaccines against capsid protein VP1 were also developed and tested in animal models. The vaccine induced a potent antitumor activity sustained by VP1-specific CD4+ and CD8+ T-lymphocytes [193].

Gambichler et al. reviewed nine clinical trials (preclinical or phase I/II) developed on vaccines in MCC in 2024. DNA- and RNA-based vaccines, along with oncolytic viruses or combinations of vaccines with immunotherapy, are detailed [194].

ACT is effective in other cancers, so Davies et al. reported a methodology for generating MCPyV TAg specific T cells. Interestingly higher responses were obtained in cells harvested from over 50 years of donors. Engineered TAg-specific CD4+ T lymphocytes secreted Th1 cytokines and induced the upregulation of CD137 when cells were challenged with MCPyV TAg peptides. Therefore, ACT for MCC patients can be another therapeutical avenue in the future [195].

Although treatment with immune checkpoint inhibitors exhibits promising outcomes in almost 50% of immunocompetent patients with MCC [196], alternative therapeutic options are essential for advanced-stage MCCs, immunocompromised patients, or patients who do not improve after classical immunotherapy [55]. Targeted molecular therapies may offer a novel breakthrough alternative that is tumor- and patient-specific. As mentioned here, each MCC subtype, whether MCPyV positive or MCPyV negative, exhibits distinct molecular characteristics that could be effectively utilized for targeted treatment.

### 6.3. Novel Therapy Approaches

#### 6.3.1. VEGF Inhibitors

As mentioned earlier, a distinct characteristic of MCC that facilitates tumor growth and metastatic spread is its high vascular density, which is supported by pro-angiogenic factors such as VEGFs.

Clinical trials of VEGF pathway inhibitors have shown promising outcomes in the treatment of neuroendocrine cancers [197] and some antiangiogenesis agents have already been approved by the FDA and are used as a standard treatment of neuroendocrine malignancies. Bob et al. studied the correlation between tumor angiogenesis and MCC clinical outcome and showed that high vascular density in MCC is associated with lower recurrence-free survival, which reinforces the hypothesis of Ng et al. that higher vascular density is a marker of worse overall MCC prognosis and survival [198,199]. In an effort to evaluate the need for targeted therapies in MCC, Brunner et al. examined 32 samples from 29 patients with MCC and found that VEGF-A, which is a proangiogenic factor, was expressed in more than 90% of them [200]. Based on these outcomes, the vascular density of MCC is considered a strong prognostic factor in its clinical outcome and can be used as a potential therapeutic target [201].

Vascular endothelial growth factor receptor (VEGFR) tyrosine kinase inhibitors (TKIs) were successfully used in the context of a clinical trial including five patients with metastatic MCCs that were previously treated with cytotoxic therapy [196]. Although limited, data are available on the use of VEGFR-TKIs, and this clinical series correlates prolonged MCC control along with adequate drug tolerance with the use of pazopanib and cabozantinib, reinforcing the hypothesis that TKIs aiming at VEGFs may provide beneficial outcomes in the treatment of disseminated MCC [196].

Imatinib, which is a TKI that reduces VEGF plasma levels and limits VEGF-independent angiogenesis [202], has been approved mainly for the treatment of hematological malignancies. It has been specifically developed to target PDGF, although it was also effective in inhibiting the BCR-ABL (chimeric fusion protein that is found in certain leukemias) and c-KIT [203]. In vitro, pharmacological inhibition of KIT with imatinib and nilotinib was associated with decreased growth of MCC-1 cells and restricted the phosphorylation of KIT [90]. Imatinib was successfully used in a large inoperable MCC in a 92-year-old patient [204] but in a phase II trial where imatinib was used as a targeted molecular therapy for MCCs that express CD117 (c-KIT) protein, little efficacy was observed, suggesting that further active and tolerable agents need to be developed for MCC [205].

#### 6.3.2. SSTs (Somatostatin Analogs)

Somatostatin is a peptide hormone known to regulate the endocrine system and cell growth, produced in many body parts, mainly exerting neuroendocrine inhibitory effects [206]. Neuroendocrine carcinomas are rich in somatostatin receptors (SSTRs), which are G protein-linked receptors that affect cell growth and proliferation, and they have become crucial therapeutical targets for multiple neuroendocrine tumors, offering an advanced treatment option [207,208].

In a recent retrospective study, SSTR2-5 expression was examined in 99 MCC tissue samples. Specifically, SSRT2 was expressed in 69% of the tumors tested, with positive staining observed in the cytoplasm and membrane. Therefore, SSRT2 cytoplasmic expression was correlated with metastatic MCCs, suggesting that SSRT2 may be a useful prognostic tool or even used as an imaging or therapeutic target [207]. Taking that into account, Buder et al. performed PET scans using somatostatin analogs in a retrospective study that included 24 patients with MCCs at various stages. SSTR-PET exhibited higher sensitivity rates than standard CT and provided a more detailed diagnostic picture of the tumor, significantly enhancing staging and altering the subsequent treatment plan [209].

Somatostatin analogs have been used in a few cases to treat advanced-stage MCC. In a case report of an 87-year-old female patient presenting with a third recurrence of MCC on her forehead, Fakiha et al. reported a successful initial response using lanreotide, which is a somatostatin analog [210]; more recently, in 2020, the use of octreotide along with avelumab is reported to have successfully treated a 73-year-old patient with recurrent MCC. In this latter case, total regression of the cancer was observed, as this was verified by a CT scan performed on a 17-month follow-up, suggesting the potential therapeutic efficacy of combined treatment with PD-1/PD-L1 immune-checkpoint inhibition and SSRTs [211]. The therapeutical efficacy of octreotide was evaluated in a recent retrospective study. Initially, 40 patients with metastatic MCCs were enrolled and SSTR expression was confirmed radiologically by somatostatin receptor scintigraphy in 19 out of them. Those patients were administered somatostatin analogs (octreotide long actin release), without concomitant use of systemic agents. Seven of them responded to treatment, while in three of them (43%), disease control was achieved over an average of 237 days [212].

Although there are only a few cases reporting the effects of somatostatin analogs, they generally have improved response rates to the treatment of MCC. Further research is needed to establish their therapeutic success, evaluate their safety, and perhaps determine potential biomarkers to evaluate the efficacy of their clinical use.

#### 6.3.3. Antivirals

Since MCPyV infection accounts for 80% of MCCs, antiviral agents could be considered as potential therapeutic options for MCPyV-positive tumors.

The MCPyV-ST and LT have an established role in the pathogenesis of MCC, counteracting against tumor-suppressing pathways, such as RB and P53, dysregulating their activity and affecting the cell cycle, thus leading to tumor growth.

Trametinib, a MEK1 and MEK2 inhibitor, was introduced by Liu et al. as an effective in vitro inhibitor of MCPyV infection [38]. Trametinib is already approved as a treatment regime for specific melanoma types, such as in the treatment of BRAFV600-mutant melanoma in combination with other agents, with good outcomes [213]. In an effort to examine the epigenetic modification enzymes and transcription factors that control MCPyV early transcription, Yang et al. investigated the effects of various epigenetic enzyme inhibitors on MCPyV-LT expression. In this context, they report that treating MCPyV-transfected cells with inhibitors against histone acetyltransferases (HATs), histone deacetylases (HDACs), and bromodomain and extra-terminal (BET) proteins, led to significantly repressed expression of MCPyV-LT [214].

Although useful only in MCPyV-positive MCCs, antiviral agents could be a useful tool. Further research in this field is required to develop effective agents that are tolerant and safe.

#### 6.3.4. PI3K Inhibitors

The PI3K/mTOR/AKT pathway is frequently aberrantly activated in both subtypes of MCC and research suggests that it has a significant implication in tumor progression. Targeting its active proteins may serve as a potential therapeutic alternative for MCC.

A case report of an 86-year-old patient with stage IV MCC, who had undergone surgery and radiotherapy, highlights the use of PI3K inhibitors. Idelalisib is a selective PI3Kδ inhibitor mainly used to treat hematologic malignancies with favorable outcomes so far. In this case report, activation of the PI3K pathway was determined in a primary cell line derived from tumors in the patient’s lymph node and overexpression of PI3Kδ was shown. Idelalisib was administered on a standard dose to the patient with good efficacy and limited side effects and complete remission was noted on a 3-month follow-up PET-CT [215].

Consistent with this data, Fang et al. evaluated the efficacy of five PI3K inhibitors with distinctive isoform-specificities. In this study, Copanlisib potently suppressed the PI3K/AKT/mTOR pathway in vitro and attenuated MCC cell proliferation and survival, hence preventing MCC colony formation. The same effect was evident in MCC xenograft tumor mouse models, where Copanlisib diminished tumor growth and stimulated apoptosis [216]. Further in vivo studies examined the efficacy of MLN0128, a second-generation dual TORC1/2 inhibitor, and outlined its markedly diminishing effect in xenograft MCC tumor growth, as well as its potent antitumor effects when administered along with JQ1 (a bromodomain protein BRD4 inhibitor). These results suggest that the PI3K/mTOR blockade could offer an effective control of MCC tumor growth [217].

Further clinical studies would be essential to establish the efficacy and safety of PI3K/mTOR/AKT targeting in the treatment of MCC, while current data suggest that a synergistic combination of PI3K/mTOR/AKT inhibitors along with other agents might offer an antitumor effect [218].

#### 6.3.5. P53 Targeting Therapeutics

The p53 signaling pathway exerts tumor suppressor effects and plays a key role in the pathogenesis of MCC. As mentioned, in MCPyV-positive MCCs, p53 is downregulated, while in MCPyV-negative MCCs, it exhibits either inactivating mutations or deletions. Houben et al. examined the possible mechanisms through which p53 is inactivated in MCC and showed that MCC-derived MCV tumor antigens do not affect the activity of p53 [67]. In the same study, Nutlin-3a, which is a specific E3 ubiquitin ligase HDM-2 inhibitor, was suggested as an alternative to cytotoxic chemotherapy [219] and used to activate p53 [67]. HDM-2 holds a key role in the negative autoregulatory loops of p53, inhibits its transcriptional activity, and keeps its levels low [220,221]. Interestingly, except MCC13, which is a p53 mutant, all five out of seven MCC cell lines demonstrated successful p53 activation [67]. Therefore, the ability of HDM-2 to reactivate p53 can be a potential therapeutic approach.

To date, research suggests that MCPyV-LT and ST interact in distinct ways with p53. MCPyV-ST increases the expression of MDM2 and CKα (which in turn interacts with MDM4), causing a downregulation in the tumor suppressive activity of p53 [65]. Milademetan is a selective MDM2 inhibitor that is orally available and affects potently the activity of MDM2. Treating MCC cell lines with wild-type p53 with Milademetan increased MDM2 levels dose-dependently and facilitated the p53 response. The same antitumor effect of Milademetan was evident in an MKL-1 xenograft model and patient-derived xenograft models, suggesting that reactivation of p53 could benefit patients with MCCs with wild-type p53 [222].

Focusing on the overexpression of MDM4 in MCPyV-positive MCC cell lines, Park et al. used Lenalidomide, which is a selective MDM4 inhibitor, which when used alone exhibited only a modest increase in p53 levels in MCC cell lines. When added to HDM201 treatment, a selective MDM2 inhibitor, Lenalidomide, substantially enhanced its efficacy in MKL-1 MCC xenografts, which allows us to hypothesize that a combination of MDM4 and MDM2 inhibition could be beneficial in MCC [65].

Although this mechanism has not been used to treat patients with MCC, preclinical data suggest that there is a strong correlation between p53 reactivation and tumor suppression. Phase I/II clinical trials (NCT03787602) on wild-type p53 MCCs were initiated but were prematurely ended.

#### 6.3.6. NOTCH Signaling Targeting Approaches

The Notch signaling pathway is frequently impaired in non-melanoma skin cancers. In MCC, some studies suggest that Notch1 membrane and cytoplasmic expression were evident in 30 out of 31 MCC cases tested [223]. UV exposure is linked with 20% of MCCs and is correlated with aberrant mutations in the Notch pathway. Given this, the effect of a recently developed agent, GP-2250, that exerts anti-tumoral activity was evaluated. Three MCPyV-negative cell lines responded adequately to GP-2250, confirming its anti-proliferative effects. Further analyses revealed that GP-2250 could downregulate Notch1 in a time- and dose-dependent manner in at least two out of three MCC cell lines, indicating that inhibition of the Notch pathway might aid in attenuating MCC [60]. The contribution of the Notch pathway in MCC still requires further investigation, in order to scrutinize its exact involvement in the pathogenesis of this tumor. Additional research would allow us to identify possible MCC therapeutic targets involved in the Notch pathway.

#### 6.3.7. MAPK/ERK-Targeting Drugs

The MARK/ERK pathway has a well-established role in cancer, with a known implication in several malignancies, where it promotes tumor progression, invasion, and resistance to therapy. Over the past decade, targeting the MAPK pathway has gained a lot of attention in cancer treatment, although “smarter”, more selective, and tolerable agents need to be developed [88]. Selective RAF/MEK/ERK inhibitors have shown remarkable results in a phase I clinical trial of patients with metastatic melanoma, with an 81% response rate [89]. In MCC dysregulation of the MAPK/ERK pathway has been observed. The JAK-STAT and MEK-ERK pathways are frequently activated in MCPyV-negative MCC resected samples, in consistency with higher activation of the MEK-ERK pathway in MCPyV-negative MCC cells, compared to MCPyV-positive cell lines. The JAK inhibitor Ruxolitinib effectively inhibited the MEK-ERK pathway in vitro, especially in MCPyV-negative MCCs, thus setting this as a potential target in MCC [224]. Additionally, the combination of MLN0128 (PI3K/AKT inhibitor), along with trametinib (MEK1/2 inhibitor), activated an interaction between PI3K/mTOR and MAPK/ERK, exhibiting synergistic effects in MCC cell lines, indicating a promising synergistic strategy [57].

#### 6.3.8. Integrins as MCC Therapy Targets

Integrins play a crucial role in the communication between the cell and the ECM. Their activity is mainly mediated by conformational changes that take place upon binding to the ECM components, activating a series of downstream intracellular signaling pathways, such as PI3K-AKT and NF-κB. Via stimulation of anti-apoptotic proteins, such as BVL-2 and FLIP, and activation of MMPs, they contribute to cancer cell migration and invasion [102,103,104]. A growing number of studies indicate that integrins also affect endothelial cell migration and survival during angiogenesis and lymphangiogenesis, which facilitate tumor growth and metastasis. Over the past decade, integrins have become an appealing target for cancer therapy due to their presence in several cell types, their contribution to cancer progression, and their interactions with growth factors [103]. Blocking integrin-mediated mechanotransduction could impair tumor cell survival and invasion.

In the preclinical setting, trials testing agents that target integrins in various malignancies have been initiated. These agents exhibit promising outcomes regarding efficacy and tolerance [225].

Very recently, a study that investigated integrin α4β1(VLA-4) as a possible target for radiopharmaceutical therapy in solid tumors was published. ITGA4, which encodes VLA-4, was overexpressed in several hematological cancer and solid tumors, and VLA-4 was expressed in 77% of the tested cancer cell lines. In the same study, PET-CT imaging with radiolabeled [67Cu]Cu-LLP2A (a peptidomimetic ligand of VLA-4) was performed, exhibiting targeted tumor dose response in a melanoma cell line, as well as in syngeneic and human cancer models. The overall low toxicity profile along with the broad expression of VLA-4 among several cancers ensures viable preclinical testing for VLA-4 as a radiopharmaceutical therapy target [226].

Little data regarding the role of integrins in MCC are available. Integrin β5 was expressed in high levels in adherent, mainly MCPyV-negative, MCC cell lines, while MCPyV itself seemed to affect integrin expression [73]. It was noted that in adherent MCC cell lines, the presence of integrin β5 and αv subunits could end up in the formation of αvβ5, which is a vitronectin receptor that is possibly essential for the attachment as well as the migration of MCC cells [73]. To date, no evidence is available on how changes in integrins’ expression or mutational alterations affect the MCC cell cycle. However, the overall implication of integrins in other cancer types is well established. A single in vivo study demonstrated that specifically targeting the αvβ3 integrin with monoclonal antibodies resulted in decreased neuroendocrine prostate tumor growth [227]. This evidence allows us to hypothesize that specific targeting of integrins may generally benefit the treatment of neuroendocrine malignancies, like MCC.

Integrins could offer a promising molecular treatment alternative in MCC treatment research. Further research on their exact role in the pathogenesis of MCCs, along with the development of potent selective inhibitors, may uncover a novel therapeutic alternative for MCCs.

#### 6.3.9. MMPs and LOX Inhibitors

Inhibitors of ECM remodeling enzymes, such as MMP inhibitors, or agents targeting ECM stiffening, such as LOX inhibitors [228], may disrupt the supportive niche required for MCC progression.

Remodeling of the ECM is pivotal in the tumor microenvironment, and MMPs are crucial ECM-modifying enzymes that can alter the synthesis of ECM, altering tissue homeostasis, affecting signaling, and hence facilitating tumor invasiveness [43]. In order to move into the bloodstream and metastasize, cancer cells must overcome histologic barriers, such as the basement membrane, stroma, and vascular basal lamina. MMPs assist these processes by degrading their molecular components, causing a turnover in the ECM [114]. MMPs also help create a friendly environment for the tumor to grow once the cancer cells have metastasized. Other than remodeling the ECM, they support neoangiogenesis around the new tumor, contribute to the release of proangiogenic growth factors, and have a crucial role in inflammation, which is strongly correlated with cancer growth [229,230,231]. Although MMPs are found on relatively low levels in normal tissues, they are overexpressed in several solid malignancies [232]. Considering all these features justifies the hypothesis that targeting MMPs could alter our approach to cancer treatment.

Several clinical trials took place between the 1980s and 2000s, testing MMP inhibitors’ efficacy against several cancer types. In contrast to preclinical research results, MMP inhibitors were not as efficient in humans and were also correlated with strong side effects [233]. Thus, Nwogu et al. presented the first attempt to examine the potential therapeutic effect of matrix metalloproteinase in MCC. In MCC, MCPyV-ST exerts augmenting effects in MMP-9, which regulates cell function [115]. Furthermore, MCPyV-ST was proven to induce the expression of EMT-associated genes such as MMP-9 and Snail, while MCPyV-LT stabilization domain (LSD) was essential in this process as it disrupts FBW7, a key regulator of MMP-9 and Snail. In vitro studies of MMP-9 targeting resulted in significantly reduced MCPyV-ST-induced cell migration and invasion, revealing a viable potential therapeutic alternative [74]. In the same context, targeting ADAM 10 and 17, which are disintegrins and metalloproteinases affecting MCC metastasis, could lower the metastatic aggressiveness of MCC, as these are promoted by MCPyV-ST [5].

MMPs generally represent exemplary targets for cancer treatment, although prior research has not had favorable outcomes. Considering that novel inhibitors have been developed over the past few years, along with modern diagnostic techniques that help us recognize tumors early, further research may reveal the antitumoral potential of MMPs [233].

Targeting the TME has generally been at the forefront of cancer treatment lately. Lysyl oxidase (LOX) is an enzyme that catalyzes the conversion of lysine residues into collagen I and elastin precursors, affecting the ECM [31]. LOX is upregulated in several malignancies and its overexpression has been associated with a worse prognosis [234]. Due to its contribution to cancer development and invasion, as well as therapy resistance, LOX inhibition has gained a lot of attention as a potential therapeutic target. A recent study highlights the potential benefits that bi-thiazole (LXG6403), a LOX inhibitor, offers in chemotherapy and ECM restructuring in cancer. In vitro and in vivo models were treated with LXG6403, which effectively reduced collagen crosslinking, restructured the ECM, and increased the chemotherapy response while also promoting DNA damage-mediated cell death [235].

#### 6.3.10. CAF-Targeting Approaches

CAFs are chief modulators of the TME that exert protumorigenic functions, facilitating cancer development. Preclinical studies have focused on targeting CAFs, exhibiting efficacy, and, hence, encouraging clinical assessment. The TME generally plays a crucial role in cancer growth and drug resistance. CAFs regulate and restructure the TME and increase protumorigenic stiffness in the ECM to assist cancer cells [236].

CAF-targeted treatments are under investigation. Reprogramming of activated CAFs into quiescent CAFs is another interesting therapeutical strategy for cancer. This alteration is characteristically mediated by All-Trans Retinoic acid (ATRA) [236]. ATRA was tested in combination with pembrolizumab, a standard PD-1 inhibitor, in stage IV melanoma patients, and the response rate and tolerability were remarkable [237]. CAFs promote cell proliferation and invasion in non-melanoma cancers. CAFs have a proven effect on the pathogenesis of MCC, mainly through regulating angiogenesis and affecting the tumor immune microenvironment [21,22]. Targeting or reprogramming their activity constitutes a bright research subject [236]. Novel therapeutic approaches are summarized in Table 2.

## 7. Future Developments

Even though recent research revealed indicative data suggesting the correlation of ECM and its components with the etiology and pathogenesis of MCC, the available evidence still remains restricted. A comprehensive investigation of the contribution of ECM in MCC pathogenesis, the identification of involved ECM components, and perhaps the identification of ECM-based biomarkers as future diagnostic or therapeutic targets may alter our approach to MCC and help us identify specific diagnostic biomarkers and innovative therapeutical targets.

## 8. Conclusions

MCC is a highly aggressive skin cancer with distinct viral and UV-induced pathogenic mechanisms that converge on common oncogenic signaling pathways. Its progression is not only driven by tumor-intrinsic alterations but also by a complex and immunosuppressive tumor microenvironment. The interplay between immune cells, ECM components, and signaling networks contributes to immune evasion and therapeutic resistance. A deeper understanding of these interactions highlights the need for combination strategies that target both tumor cells and the surrounding microenvironment to improve treatment efficacy and long-term outcomes in MCC.

## Figures and Tables

**Table 1:** A summary of the identified signaling pathways in MCC pathogenesis.

Signaling Pathway	Involvement in MCC
PI3K/AKT/mTOR	-Activating mutations in PI3KCA identified in MCC [49]; -AKT phosphorylation at threonine 308 [50]; -Screening for *PIK3CA* mutations could help identify patients who might benefit from PI3K pathway inhibitors [51]; -MCPyV-negative MCCs exhibit a higher p-AKT activation frequency [52]; -Genes upregulated in deceased patients were primarily associated with the PI3K/AKT pathway [53]; -Inhibition of AKT results in the inactivation of mTOR and glycogen synthase kinase 3 pathway proteins, upregulation of p16 expression, and modulation of the phosphorylation of the B-cell lymphoma-2-associated death promoter, leading to suppression of MCC cell proliferation [54]; -Chemical dual mTORC1/mTORC2 inhibition suppresses MCC cell growth in vitro and in vivo [55].
MAPK/ERK	-A subset of genes upregulated in deceased MCC patients was linked to MAPK pathway activation, independently of the viral status [52]; -A gene expression profiling study identified 2 distinct molecular subtypes of MCC, where Subtype II is associated with overexpression of genes involved in the TNF signaling and MAPK signaling pathways [56]; -MCPyV-ST activates p38 MAPK signaling, driving cell migration and motility [57]; Genes upregulated in deceased patients were primarily associated with angiogenesis and the MAPK pathway [53].
Notch	-NOTCH1 membrane and cytoplasmic expression identified in MCC [58]; -NOTCH1 and NOTCH2 expression is not correlated with MCPyV status or prognosis [59]; -NOTCH3 expression is higher in MCPyV-positive MCCs and it is associated with enhanced prognosis (NOTCH3 expression serves as a prognostic MCC marker) [59]; -Expression of JAG1 (Notch ligand) is higher in MCPyV-negative MCCs [59]; -Decreased Notch expression correlated with increased necrosis and apoptosis of MCPyV-negative tumor cells [60]; -Higher expression of EMT-related genes is observed in MCPyV-negative MCCs, which were enriched in Notch signaling [61].
TP53	-MCPyV-negative MCCs present inactivating mutations or deletions of TP53 [12]; -MCPyV-positive MCCs exhibit downregulated activity of TP53 [12]; -UVB-specific mutations were identified in the p53 pathway [62,63]; -TP53 mutations mainly present in MCPyV-DNA-negative MCCs [64]; -The p53 pathway is activated in Positive MCCs via the binding of MCPyV-LT to pRB, while the MCPyV-ST downregulates p53 by increasing the levels of MDM2 and CKα [65]; -p53 inactivating mutations are generally low in MCCs (10–14%) [66,67].
RB	-MCPyV-LT targets pRB and binds strongly and inactivates RB1, promoting tumor growth [68]; -RB1 gene copy loss found on over 60% of MCPyV-negative MCCs [69]; -In a study incuding 13 MCPyV-positive and 13 MCPyV-negative tumors, RB expression was significantly higher in MCPyV-positive MCCs and was associated with better prognosis [70]; -The RB1 promoter is hypermethylated in all MCCs, regardless of their RB expression [70].
Wnt/β-Catenin	-UV exposure along with advanced age triggers the Wnt/β-catenin pathway [38]; -Accumulation of β-catenin is infrequent in MCC [71,72].
Integrins	-Identified in MCC cell line-derived exosomes [38]; -Integrin β1 is involved in MCC filopodia formation [35]; -Integrin β5 is highly expressed in adherent MCC cell lines, which are mainly MCPyV-negative [73].
ECM Remodeling Enzymes	-MCPyV-ST enhances MMP-9 and Snail, while MMP9 inhibition reduces MCPyV-ST-driven cell migration and invasion [74]; -WNT/β-catenin signaling pathway and other growth factors induce MMP1, MMP3, MMP7, MMP9, MMP10, MMP11, and MMP13 gene expression, which in turn enhances MCPyV infection [38]; -High levels of MMP7 and MMP10/2 correlate with MCC’s metastatic capacity [40]; -Distinct expression patterns of MMP-10, MMP-21, MMP-26, and MMP-28 have been identified in primary MCCs and lymph node metastases. MMP-28 was found in tumor cells, particularly in smaller tumors (<2 cm) [39]; -MMP26 was expressed in stromal cells, associated with larger tumors (>2cm) and poor prognosis [39]; -MMP10 is the most frequently expressed and prominent in metastatic lymph nodes matrix metalloproteinase [39].

**Table 2:** Novel therapeutic approaches.

Therapy Targeting Small Molecule Inhibitors	Concept and Available Evidence
VEGF inhibitors	Antiangiogenesis agents have already been approved for the treatment of several neuroendocrine malignancies [197]; High vascular density in MCC has been associated with worse prognosis and survival [198,199]; In vitro inhibition of KIT shows promising results [90]; VEGFR TKIs have been used against MCC in limited clinical trials with controversial outcomes [196].
2. SSTs	Neuroendocrine carcinomas are rich in SSTRs [207,208]; SSTR2 cytoplasmic expression is associated with metastatic MCCs [207]; SSTs have been used with or without immune checkpoint inhibitors in few cases, with successful outcomes [210,211,212].
3. Antivirals	MCPyV infection accounts for 8 out of 10 MCCs [7]; MCPyV-LT expression was significantly suppressed by various epigenetic enzyme inhibitors (e.g., inhibitors against HATs, HDACs, BET proteins) [214].
4. PI3K inhibitors	Both MCC subtypes have been associated with PI3K/mTOR/AKT pathway abberant activation [52]; In vitro and in vivo studies show promising results regarding the effect of PI3K inhibitors against MCC tumor growth and metastasis [216,217].
5. P53	The MCPyV-ST and LT interact with p53 in distinct pathways [65,99]; Data suggest that p53 reactivation may support MCC tumor suppression [222]; Phase I/II clinical trials on wild type p53 MCCs are currently taking place
6. NOTCH	Impairment of the Notch pathway is frequent in non-melanoma skin cancers [95]; UV exposure, which is accountable for 20% of MCCs, alters the normal Notch pathway [60]; In vitro studies suggest that downregulation of Notch1 could offer promising outcomes in the treatment of MCC [60].
7. MAPK/ERK	MAPK/ERK dysregulation has been observed in MCCs [53]; In vitro JAK inhibitors successfully inhibited the MEK-ERK pathway, mainly in MCPyV-negative MCCs [224].
8. Integris	Altered integrin expression has been observed in MCPyV-negative MCC cell lines [73]; Targeting integrins with monoclonal antibodies has been tested in other neuroendocrine malognancies, such as neuroendocrine prostate cancer [227].
9. MMPs and LOX	MMPs are crucial in ECM remodeling, facilitating tumor growth [43]; In vitro studies of MMP-9 targeting significantly reduced the MCPyV-ST-induced cell migration and invasion [74]; LOX targeting enhances the chemoresponse and ECM restructuring in cancer, as shown by in vitro and in vivo studies [235].
10. CAFs	CAFs have a key role in remodeling and restructuring the TME [236]; CAFs have a proven crucial contribution in the pathogenesis of MCC [21,22]; Targeting or reprogramming CAFs may offer a promising therapeutical approach in MCC, although further research remains essential [236].

## Abbreviations

Acronym	Abbreviation
MCC	Merkel Cell Carcinoma
MCPyV	Merkel Cell Polyomavirus
UV exposure	Ultraviolet exposure
pRb	Retinoblastoma protein
P53	Tumor protein 53
PI3K	Phosphatidylinositol-3-kinase
AKT	Protein kinase B
mTOR	Mammalian target of rapamycin
MAPK	Mitogen-activated protein kinase
TME	Tumor Microenvironment
PD-1	Programmed cell death 1
PD-L1	Programmed death - ligand 1
ECM	Extracellular matrix
MMPs	Matrix metalloproteinases
AJCC	American Joint Cancer Committee
CAFs	Cancer associated fibroblasts
GAGs	Glycosaminoglycans
PGs	Proteoglycans
LOX	Lysyl oxidase
Tn-C	Tenascin-C
TLR4	Toll-like receptor 4
FAK	Focal adhesion kinase
YAP	Yes-associated protein
TGFβ	Transforming growth facto beta
PDGF	Platelet-derived growth factor
FGF	Fibroblast growth factor
HGF	Hepatocyte growth factor
VEGF	Vascular endothelial growth factor
TNF	Tumor necrosis factor
IFN	Interferon
CXCL12	C-X-C motif chemokine ligand 12
IL-6	Interleukin-6
CTGFβ	Connective tissue growth factor
EGF	Epidermal growth factor
GAS6	Growth arrest-specific protein 6
SFRP1	Galectin-1, secreted frizzled-related protein 1
SHH	Sonic hedgehog protein
BMP	Bone morphogenetic protein
scRNAseq	Single-cell RNA sequencing
miR-375	MicroRNA-375
RBPJ	Recombination signal binding protein for immunoglobulin kappa J region
CD8	Cluster of differentiation 8
CD4	Cluster of differentiation 4
TILs	Tumor-infiltrating Lymphocytes
HIFs	Hypoxia-inducible factors
αSMA-1+ cells	α Smooth muscle actin positive stromal cells
CD31	Cluster of differentiation 31
B7-H3	B7 homolog 3 protein
VEGFR	Vascular endothelial growth factor receptor
RTKs	Receptor tyrosine kinases
GPCRs	G-protein-coupled receptors
PIP2	Phosphatidylinositol-4,5-bisphosphate
PIP3	Phosphatidylinositol-3,4,5-trisphosphate
FOXO	Forkhead box transcription factors
PTEN	Phosphatase and tensin homolog
TMB	Tumor mutation burden
BRCA	Breast Cancer gene
HRAS	Harvey rat sarcoma viral oncogene homolog
TSC	Tuberous sclerosis proteins
ARID1A	AT-Rich Interaction Domain 1A
CDKN2A	Cyclin-dependent kinase inhibitor 2A
NOTCH1	Neurogenic locus notch homolog protein 1
MEK-ERK	Mitogen-activated protein kinase/extracellular-signal-regulated kinase
JAK-STAT	Janus kinase/signal transducer and activator of transcription
SCF	stem cell factor
MKK4	Mitogen-activated protein kinase (MAPK) kinase 4
NICD	Notch intracellular domain
TA	Tumor antigens
HDM-2	Human double minute 2
BCL-2	B-cell lymphoma-2 protein
FLIP	Fas-associated death domain-like interleukin-1-β-converting enzyme-inhibitory protein
NF-κB	Nuclear factor kappa-light-chain-enhancer of activated B cells
ST	Small tumor antigen
LT	Large tumor antigen
PP4C	Phosphatase catalytic subunit
PIEZO	Mechanosensitive ion channels located in the cell membrane and function as key cellular mechanotransducers
GPCR	G protein-coupled receptor
IHC	Immunohistochemistry
HLA	Human leukocyte antigens
aAPC	Artificial antigen-presenting
ISGF3	Interferon-stimulated gene factor 3
TAM	Tumor-associated macrophages
APC	Antigen-presenting cells
NFAT	Nuclear factor of activated T cells
NFP	Neurofilament
TTF-1	Thyroid transcription factor 1
SLNB	Sentinel Lymph Node Biopsy
TCM	Central memory T cells
ROS	Reactive oxygen species
STING	Stimulator of interferon genes
TCR	T-cell receptor
DFs	Dermal fibroblasts
ACT	Adoptive T cell transfer
PET	Positron Emission Tomography-Computed Tomography
HATs	Histone acetyltransferases
HDACs	Histone deacetylases
BET	Bromodomain and extra-terminal
